# Medicinal Plants in the Prevention and Treatment of Colon Cancer

**DOI:** 10.1155/2019/2075614

**Published:** 2019-12-04

**Authors:** Paola Aiello, Maedeh Sharghi, Shabnam Malekpour Mansourkhani, Azam Pourabbasi Ardekan, Leila Jouybari, Nahid Daraei, Khadijeh Peiro, Sima Mohamadian, Mahdiyeh Rezaei, Mahdi Heidari, Ilaria Peluso, Fereshteh Ghorat, Anupam Bishayee, Wesam Kooti

**Affiliations:** ^1^Council for Agricultural Research and Economics, Research Centre for Food and Nutrition, Via Ardeatina 546, 00178 Rome, Italy; ^2^Department of Physiology and Pharmacology “V. Erspamer”, La Sapienza University of Rome, Rome, Italy; ^3^Nursing and Midwifery School, Guilan University of Medical Sciences, Rasht, Iran; ^4^Department of Biology, School of Science, Shiraz University, Shiraz, Iran; ^5^Lung Diseases and Allergy Research Center, Research Institute for Health Development, Kurdistan University of Medical Sciences, Sanandaj, Iran; ^6^Nursing Research Center, Golestan University of Medical Sciences, Gorgan, Iran; ^7^Student Research Committee, Ahvaz Jundishapur University of Medical Sciences, Ahvaz, Iran; ^8^Department of Biology, Faculty of Sciences, Shahid Chamran University, Ahvaz, Iran; ^9^Faculty of Pharmacy and Pharmaceutical Sciences, Tehran Medical Sciences, Islamic Azad University, Tehran, Iran; ^10^Traditional and Complementary Medicine Research Center, Sabzevar University of Medical Sciences, Sabzevar, Iran; ^11^Lake Erie College of Osteopathic Medicine, 5000 Lakewood Ranch Boulevard, Bradenton, FL 34211, USA

## Abstract

The standard treatment for cancer is generally based on using cytotoxic drugs, radiotherapy, chemotherapy, and surgery. However, the use of traditional treatments has received attention in recent years. The aim of the present work was to provide an overview of medicinal plants effective on colon cancer with special emphasis on bioactive components and underlying mechanisms of action. Various literature databases, including Web of Science, PubMed, and Scopus, were used and English language articles were considered. Based on literature search, 172 experimental studies and 71 clinical cases on 190 plants were included. The results indicate that grape, soybean, green tea, garlic, olive, and pomegranate are the most effective plants against colon cancer. In these studies, fruits, seeds, leaves, and plant roots were used for *in vitro* and *in vivo* models. Various anticolon cancer mechanisms of these medicinal plants include induction of superoxide dismutase, reduction of DNA oxidation, induction of apoptosis by inducing a cell cycle arrest in S phase, reducing the expression of PI3K, P-Akt protein, and MMP as well; reduction of antiapoptotic Bcl-2 and Bcl-xL proteins, and decrease of proliferating cell nuclear antigen (PCNA), cyclin A, cyclin D1, cyclin B1 and cyclin E. Plant compounds also increase both the expression of the cell cycle inhibitors p53, p21, and p27, and the BAD, Bax, caspase 3, caspase 7, caspase 8, and caspase 9 proteins levels. In fact, purification of herbal compounds and demonstration of their efficacy in appropriate *in vivo* models, as well as clinical studies, may lead to alternative and effective ways of controlling and treating colon cancer.

## 1. Introduction

An uncontrolled growth of the body's cells can lead to cancer. Cancer of the large intestine (colon) is one of the main cause of death due to cancer. While the numbers for colon cancer are somewhat equal in women (47,820) and men (47,700), it will be diagnosed in (16,190) men (23,720) more than women. Multiple factors are involved in the development of colorectal cancer, such as lack of physical activity [1], excessive alcohol consumption [2], old age [3], family history [4], high-fat diets with no fiber and red meat, diabetes [5], and inflammatory bowel diseases, including ulcerative colitis and Crohn's disease [6].

Prevention of colorectal cancer usually depends on screening methods to diagnose adenomatous polyps which are precursor lesions to colon cancer [7]. The standard treatment for cancer is generally based on using cytotoxic drugs, radiotherapy, chemotherapy, and surgery [8]. Apart from these treatments, antiangiogenic agents are also used for the treatment and control of cancer progression [9].

Colon cancer has several stages: 0, I, II, III, and IV. Treatment for stages 0 to III typically involves surgery, while for stage IV and the recurrent colon cancer both surgery and chemotherapy are the options [10]. Depending on the cancer stage and the patient characteristics, several chemotherapeutic drugs and diets have been recommended for the management of colorectal cancer. Drugs such as 5-fluorouracil (5-FU), at the base of the neoadjuvant therapies folfox and folfiri, are used together with bevacizumab, panitumumab, or cetuximab [7].

Chemotherapy works on active cells (live cells), such as cancerous ones, which grow and divide more rapidly than other cells. But some healthy cells are active too, including blood, gastrointestinal tract, and hair follicle ones. Side effects of chemotherapy occur when healthy cells are damaged. Among these side effects, fatigue, headache, muscle pain, stomach pain, diarrhea and vomiting, sore throat, blood abnormalities, constipation, damage to the nervous system, memory problems, loss of appetite, and hair loss can be mentioned [11].

Throughout the world, early diagnosis and treatment of cancer usually increase the individual's chances of survival. But in developing countries, access to effective and modern diagnostic methods and facilities is usually limited for most people, especially in rural areas [12]. Accordingly, the World Health Organization (WHO) has estimated that about 80% of the world population use traditional treatments [13]. One of these treatments is phytotherapy, also known as phytomedicine, namely, the use of plants or a mixture of plant extracts for the treatment of diseases. The use of medicinal plants can restore the body's ability to protect, regulate, and heal itself, promoting a physical, mental, and emotional well-being [14–16]. Various studies have shown the therapeutic effects of plants on fertility and infertility [17], hormonal disorders, hyperlipidemia [18], liver diseases [19], anemia [20], renal diseases [21], and neurological and psychiatric diseases [22]. Therefore, due to all the positive effects showed by medicinal plants, their potential use in cancer prevention and therapy has been widely suggested [23–25].

Since the current treatments usually have side effects, plants and their extracts can be useful in the treatment of colon cancer with fewer side effects. The aims of this review are to present and analyse the evidence of medicinal plants effective on colon cancer, to investigate and identify the most important compounds present in these plant extracts, and to decipher underlying molecular mechanisms of action.

## 2. Literature Search Methodology

This is a narrative review of all research (English full text or abstract) studies conducted on effective medicinal plants in the treatment or prevention of colon cancer throughout the world. Keywords, including colon cancer, extract, herbs, plant extracts, and plants, were searched separately or combined in various literature databases, such as Web of Science, PubMed, and Scopus. Only English language articles published until July 2018 were considered.

In the current narrative review, studies (published papers) were accepted on the basis of inclusion and exclusion criteria. The inclusion criterion was English language studies, which demonstrated an effective use of whole plants or herbal ingredients, as well as studies which included standard laboratory tests. *In vivo* and *in vitro* studies that were published as original articles or short communications were also included. The exclusion criteria included irrelevancy of the studies to the subject matter, not sufficient data in the study, studies on mushrooms or algae, and the lack of access to the full text. Reviews, case reports/case series, and letters to editors were also excluded but used to find appropriate primary literature.

The abstracts of the studies were reviewed independently by two reviewers (authors of this study) on the basis of the inclusion and exclusion criteria. In case of any inconsistency, both authors reviewed the results together and solved the discrepancy. Data extracted from various articles were included in the study and entered into a check list after the quality was confirmed. This check list included some information: authors' name, year of publication, experimental model, type of extract and its concentration or dose, main components, and mechanisms of action (if reported).

## 3. Results

### 3.1. Medicinal Plants and Colon Cancer

Overall, 1,150 articles were collected in the first step and unrelated articles were excluded later on according to title and abstract evaluation. Moreover, articles that did not have complete data along with congress and conference proceedings were excluded. Accordingly, a total of 1,012 articles were excluded in this step. Finally, 190 articles fulfilled the criteria and were included in this review. These papers were published within 2000-2017. A total of 190 plants were included in this study. Based on literature search, 172 experimental studies and 71 clinical cases were included.

Overall, results indicate that grape, soybean, green tea, garlic, olive, and pomegranate are the most effective plants against colon cancer. In these studies, fruits, seeds, leaves, and plant roots were used for *in vitro* and *in vivo* studies.

#### 3.1.1. *In Vitro* Studies

Out of 172 studies, 75 were carried out on HT-29, 60 on HCT116, and 24 on Caco-2 cells (Table 1). On HT-29 cells, both *Allium sativum* root extracts and *Camellia sinensis* leaf extracts induced cell apoptosis by two different mechanisms, respectively. In fact, the former showed inhibition of the PI3K/Akt pathway, upregulation of PTEN, and downregulation of Akt and p-Akt expression, while the latter was involved in attenuation of COX-2 expression and modulation of NF*κ*B, AP-1, CREB, and/or NF-IL-6. Moreover, an antiproliferative activity has also been detected in *Olea europaea* fruit extracts, which increased caspase 3-like activity and were involved in the production of superoxide anions in mitochondria. An antiproliferative activity, by means of a blockage in the G2/M phase, has also been reported in Caco-2 cells by *Vitis vinifera* fruit extracts. Concerning HCT116 cells, several plants, such as *American ginseng* and *Hibiscus cannabinus*, induced cell cycle arrest in different checkpoints.

#### 3.1.2. Studies in Animal Models

The most used animal model is the murine one (Tables 2(a) and 2(b)). In particular, studies were carried out above all on HT-29 and HCT116 cells. The effects of the different medicinal plants and their extracts are essentially the same detected in *in vitro* studies. In particular, plant extracts were able to induce apoptosis and inhibit proliferation and tumor angiogenesis by regulating p53 levels and checkpoint proteins with consequent cell cycle arrest and antiproliferative and antiapoptotic effects on cancerous cells.

**Figure 1:**
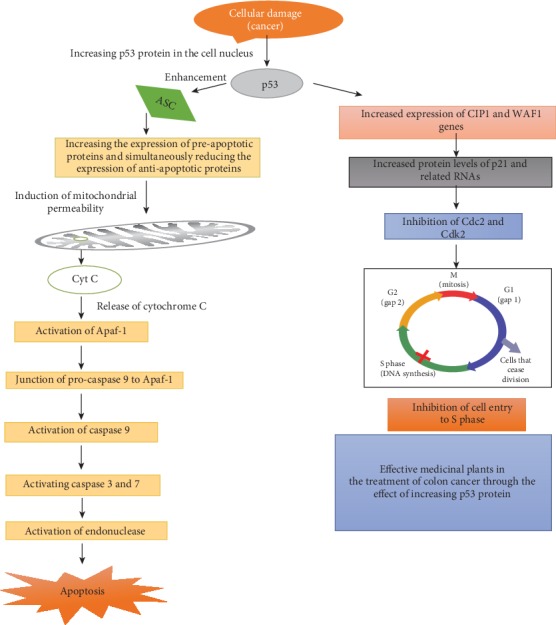
Cell damage and cancer trigger p53 activation. The p53 protein activates the apoptotic protein Bax. Bax inhibits the antiapoptotic protein Bcl-2. During apoptosis, cytochrome c is released from mitochondria. To activate the Apaf-1 protein, the interaction between these proteins and cytochrome C is necessary. Pro-caspase 9 attaches to Apaf-1 and activates caspase 9. Caspase 9 activates caspases 3 and 7 and apoptosis occurs.

The main mechanisms of action of medicinal plants are summarized in Figure 1.

In *in vitro* studies, it has been found that grapes, which contain substantial amounts of flavonoids and procyanidins, play a role in reducing the proliferation of cancer cells by increasing dihydroceramides and p53 and p21 (cell cycle gate keeper) protein levels. Additionally, grape extracts triggered antioxidant response by activating the transcriptional factor nuclear factor erythroid 2-related factor 2 (Nrf2) [27].

Grape seeds contain polyphenolic and procyanidin compounds, and their reducing effects on the activity of myeloperoxidase have been shown in *in vitro* and *in vivo* studies. It has been suggested that grape seeds could inhibit the growth of colon cancer cells by altering the cell cycle, which would lead eventually to exert the caspase-dependent apoptosis [180].

Another plant that attracted researchers' attention was soybean, which contain saponins. After 72 h of exposure of colon cancer cells to the soy extract, it was found that this extract inhibited the activity and expression of protein kinase C and cyclooxygenase-2 (COX-2) [34]. The density of the cancer cells being exposed to the soy extract significantly decreased. Soybeans can also reduce the number of cancer cells and increase their mortality, which may be due to increased levels of Rab6 protein [216].

Green tea leaves have also attracted the researchers' attention in these studies. Green tea leaves, with high levels of catechins, increased apoptosis in colon cancer cells and reduced the expression of the vascular endothelial growth factor (VEGF) and its promoter activity in *in vitro* and *in vivo* studies. The extract increased apoptosis (programmed cell death) by 1.9 times in tumor cells and 3 times in endothelial cells compared to the control group [182]. In another *in vitro* study, the results showed that green tea leaves can be effective in the inhibition of matrix metalloproteinase 9 (MMP-9) and in inhibiting the secretion of VEGF [183].

Garlic was another effective plant in this study. Its roots have allicin and organosulfur compounds. In an *in vitro* study, they inhibited cancer cell growth and induced apoptosis through the inhibition of the phosphoinositide 3-kinase/Akt pathway. They can also increase the expression of phosphatase and tensin homolog (PTEN) and reduce the expression of Akt and p-Akt [32]. Garlic roots contain S-allylcysteine and S-allylmercaptocysteine, which are known to exhibit anticancer properties. The results of a clinical trial on 51 patients, whose illness was diagnosed as colon cancer through colonoscopy, and who ranged in age from 40 to 79 years, suggest that the garlic extract has an inhibitory effect on the size and number of cancer cells. Possible mechanisms suggested for the anticancer effects of the garlic extract are both the increase of detoxifying enzyme soluble adenylyl cyclase (SAC) and an increased activity of glutathione S-transferase (GST). The results suggest that the garlic extract stimulates mouse spleen cells, causes the secretion of cytokines, such as interleukin-2 (IL2), tumor necrosis factor-*α* (TNF-*α*), and interferon-*γ*, and increases the activity of natural killer (NK) cells and phagocytic peritoneal macrophages [200].

The results of *in vitro* studies on olive fruit showed that it can increase peroxide anions in the mitochondria of HT-29 cancer cells due to the presence of 73.25% of maslinic acid and 25.75% of oleanolic acid. It also increases caspase 3-like activity up to 6 times and induces programmed cell death through the internal pathway [217]. Furthermore, the olive extract induces the production of reactive oxygen species (ROS) and causes a quick release of cytochrome c from mitochondria to cytosol.

The pomegranate fruit contains numerous phytochemicals, such as punicalagins, ellagitannins, ellagic acid, and other flavonoids, including quercetin, kaempferol, and luteolin glycosides. The results of an *in vitro* study indicate the anticancer activity of this extract through reduction of phosphorylation of the p65 subunit and subsequent inhibition of nuclear factor-*κ*B (NF*κ*B). It also inhibits the activity of TNF receptor induced by Akt, which is needed for the activity of NF*κ*B. The fruit juice can considerably inhibit the expression of TNF-*α*-inducing proteins (Tip*α*) in the COX-2 pathway in cancer cells [43]. The effective and important compounds in pomegranate identified in these 104 studies are flavonoids, polyphenol compounds, such as caffeic acid, catechins, saponins, polysaccharides, triterpenoids, alkaloids, glycosides, and phenols, such as quercetin and luteolin, and kaempferol and luteolin glycosides.

In a systematic review of the plants being studied, some mechanisms were mainly common, including the induction of apoptosis by means of an increase of expression and levels of caspase 2, caspase 3, caspase 7, caspase 8, and caspase 9 in cancer cells, increasing the expression of the proapoptotic protein Bax and decreasing the expression of the antiapoptotic proteins.

Many herbal extracts block specific phase of the cell cycle. For instance, the extract prepared from the leaves of *Annona muricata* inhibits the proliferation of colon cancer cells and induces apoptosis by arresting cells in the G1 phase [53]. They can also prevent the progress of the G1/S phase in cancer cells [74]. In general, the herbal extracts reported here have been able to stop cancer cells at various stages, such as G2/M, G1/S, S phase, G0/G1, and G1 phase, and could prevent their proliferation and growth.

Other important anticancer mechanisms are the increase of both p53 protein levels and transcription of its gene. Even the increase of p21 expression is not without effect [137]. In an *in vitro* study on the *Garcinia mangostana* roots, the results were indicative of the inhibitory effect of the extract of this plant on p50 and P65 activation [93]. Moreover, reduction of cyclin D1 levels and increase of p21 levels are among these mechanisms [137], as well as inhibition of NF*κ*B and reduction of the transcription of its genes, which contribute to reduce the number of cancerous cells [127]. Other important anticancer mechanisms are the inhibition of COX-2, as well as the reduction of the protein levels in this pathway [34]. In addition to this, in some cases, the inhibition of MMP-9 can be mentioned as the significant mechanism of some herbal extracts to kill cancer cells [183].

## 4. Conclusion and Perspectives

The findings of this review indicate that medicinal plants containing various phytochemicals, such as flavonoids, polyphenol compounds, such as caffeic acid, catechins, saponins, polysaccharides, triterpenoids, alkaloids, glycosides, and phenols, such as quercetin and luteolin, and kaempferol and luteolin glycosides, can inhibit tumor cell proliferation and also intduce apoptosis.

Plants and their main compounds affect transcription and cell cycle via different mechanisms. Among these pathways, we can point to induction of superoxide dismutase to eliminate free radicals, reduction of DNA oxidation, induction of apoptosis by inducing a cell cycle arrest in S phase, reduction of PI3K, P-Akt protein, and MMP expression, reduction of antiapoptotic Bcl-2, Bcl-xL proteins, and decrease of proliferating cell nuclear antigen (PCNA), cyclin A, cyclin D1, cyclin B1, and cyclin E. Plant compounds also increase the expression of both cell cycle inhibitors, such as p53, p21, and p27, and BAD, Bax, caspase 3, caspase 7, caspase 8, and caspase 9 proteins levels. In general, this study showed that medicinal plants are potentially able to inhibit growth and proliferation of colon cancer cells. But the clinical usage of these results requires more studies on these compounds in *in vivo* models. Despite many studies' *in vivo* models, rarely clinical trials were observed among the studies. In fact, purification of herbal compounds and demonstration of their efficacy in appropriate *in vivo* models, as well as clinical studies, may lead to alternative and effective ways of controlling and treating colon cancer.

## Figures and Tables

**Table 1:** Cytotoxic effects of medicinal plants on colon cancer in *in vitro* models.

Scientific name	Parts used	Cell line	Conc.	Type of extract	Important compounds	Cellular effect	Mechanisms	References
*Vitis vinifera*	Fruit	HCT116	NM	*Lyophilized*	Hydroxycinnamic acids, proanthocyanidins, stilbenoids	Increase of dihydroceramides, sphingolipid mediators involved in cell cycle arrest, and reduction of the proliferation rate	(i) Increase of p53 and p21 cell cycle gate keepers (ii) Activation of the transcriptional factor Nrf2	[26, 27]
	Fruit	Caco-2	365 mg/g	*Methanolic*	Catechin, epicatechin, quercetin, gallic acid	Antiproliferative activity and direct initiation of cell death	Blockage in the G2/M phase	[28, 29]
	Seed	Caco-2	10–25 *μ*g/mL	*Aqueous*	Procyanidins	(i) Increased crypt depth (ii) Inhibited cell viability and decreased histological damage score	Reduced MPO (myeloperoxidase) activity	[29]
	Skin	NM	7.5, 30, 60 *μ*g/mL	*Methanolic*	4′-Geranyloxyferulic acid	NM	NM	[30]
	Seed	Colon cancer stem cells	6.25, 12.5, 25 *μ*g/mL	*NM*	(+)-catechin, (−)-epicatechin	NM	(i) Increment of p53, Bax/Bcl-2 ratio, and cleaved PARP (ii) Inhibition of Wnt/*β*-catenin signaling	[31]

*Allium sativum*	Root	HT-29	20, 50, 100 mg/mL	*Ethanolic*	NM	Induction of apoptosis and cell cycle arrest	(i) Inhibition of the PI3K̸Akt pathway (ii) Upregulation of PTEN and downregulation of Akt and p-Akt expression	[32]

*Glycine max*	Seed	Caco-2, SW620, HT-29	12.5 *μ*g/mL	*Aqueous*	Anthoxanthin	Cell death and significant reduction of cell density	Enhancement of Rab6 protein levels	[33]
	Seed	HT-29	240, 600 ppm	*Crude*	Saponin		Suppression of PKC activation and increase of alkaline phosphatase activity	[33]
	Seed	HT-29	NM	*Crude*	Saponin	NM	(i) Suppression of the degradation of I*κ*B*α* in PMA-stimulated cells (ii) Downregulation of COX-2 and PKC expressions	[34]

*Camellia sinensis*	Leaf	HT-29	0, 10, 30, 50 *μ*M	*Aqueous*	Catechin, epigallocatechin gallate	1.9-fold increase in tumor cell apoptosis and a 3-fold increase in endothelial cell apoptosis	(i) Suppression of ERK-1 and ERK-2 activation (ii) Suppression of VEGF expression	[35]
	Leaf	Caco-2, HT-29	300 *μ*M	*Aqueous*	Theaflavins (TF-2T, F-3, TF-1)	Human colon cancer cell apoptosis induction	Modulation of NF*κ*B, AP-1, CREB, and/or IL-6	[36]
	Leaf	HT-29	68-80 0.73 *μ*g/mL	*Hot water extract*	Flavan-3-ol (catechin & tannin) & polyphenols (teadenol B)	Inhibition of proliferation of HT-29 cells	Increased expression levels of caspases 3/7, 8, and 9	[35]

*Olea europaea*	Fruit	HT-29	150, 55.5 200 and 74 *μ*mol/L	*Methanolic and chloroform*	Maslinic acid, oleanolic acid	Antiproliferative activity	(i) Increased caspase 3-like activity to 6-fold (ii) Production of superoxide anions in the mitochondria	[37]
	Fruit, leaf	SW480 and HT-29	100–400 m/z	*Methanolic & hexane*	Oleic acid, linoleic acid, gamma-linolenic acid, lignans, flavonoids, secoiridoids	Reduced cell growth in both cell lines	(i) Limited G2M cell cycle (ii) Depressed cyclooxygenase-2 expression in HT-29 cells (iii) Suppression of *β*-catenin/TCF signaling in SW480 cells (iv) Promotion of the entry into subG1 phase	[38]
	Fruit	Caco-2	50 *μ*M	*Aqueous*	Phenolic compounds, authentic hydroxyl tyrosol (HT)	Reduced proliferation of Caco-2 cells	Reduction of the methylation levels of CNR1 promoter	[39]
	Fruit	HT115	25 *μ*g/mL	*Hydroethanolic*	Phenolic compounds (p-hydroxyphenyl ethanol, pinoresinol & dihydroxyphenyl ethanol)	NM	Inhibition by reduced expression of a range of *α*5 & *β*1	[40]
	Olive mill wastewater	HT-29, HCT116, CT26	NM	*Methanolic*	Hydroxytyrosol	(i) Inhibited proliferation (ii) Inhibited migration and invasion	(i) Reduced sprout formation (ii) Inhibited VEGF and IL-8 levels	[41]
	Fruit	Caco-2	0-2,000 *μ*g/mL	*Ethanolic*	Tyrosol, hydroxytyrosol, oleuropein, rutin, quercetin and glucoside forms of luteolin and apigenin	NM	(i) Induction of the cell cycle arrest in S-phase	[42]

*Punica granatum*	Juice	HT-29	50 mg/L	*Aqueous*	Ellagitannins, punicalagin	Inhibition of cancer cell proliferation	(i) Suppressed TNFR-induced COX-2 protein expression (ii) Reduced phosphorylation of the p65 subunit and binding to the NF*κ*B response element	[43]
	Seed	LS174	63.2 *μ*g/mL	*Supercritical fluid*	Punicic acid, *γ*-tocopherol, *α*-tocopherol	Cytotoxic activity	(i) Slightly decreased development of tubules from elongated cell bodies (ii) Reduction of the number of cell connections	[44]

*Glycyrrhiza glabra*	Root	HT-29	12.2 and 31 *μ*g/mL	*Ethanolic*	Licochalcone	NM	Increase of the protein levels of proapoptotic Bax	[37]

*Opuntia ficus-indica*	Fruit	Caco-2	115 *μ*M	*Aqueous*	Betalain pigment indicaxanthin	Apoptosis of proliferating cells	(i) Demethylation of the tumor suppressor p16INK4a gene promoter (ii) Reactivation of the silenced mRNA expression and accumulation of p16INK4a	[38]
	Fruit	HT-29 & Caco-2 & NIH 3 T3 (as control)	Against HT-29 (4.9 *μ*g/mL) against Caco-2 (8.2 *μ*g/mL)	*Alkaline hydrolysis with NaOH*	Isorhamnetin glycosides (IG5 and IG6)-phenol	Cell death through apoptosis and necrosis	Increased activity of caspase 3/7	[45]

*Piper betle*	Leaf	HT-29 and HCT116	200.0 *μ*g/mL	*Aqueous*	Hydroxychavicol	Antioxidant capacity and induction of a greater apoptotic effect	(i) Scavenging activity (ii) Formation of electrophilic metabolites	[41, 46]

*Fragaria×ananassa*	Fruit	HT-29	0.025, 0.05, 0.25, 0.5%	*Ethanolic*	Ascorbate, ellagic acid	Decreased proliferation of HT-29 cells	Increase in the levels of 8OHA and decrease in the levels of 8OHG	[40]

*Sasa quelpaertensis*	Leaf	HT-29 HCT116	0, 100, 200, 300 mg/L	*Ethanolic*	p-Coumaric acid, tricin	Inhibited colony formation	Nonadherent sphere formation suppressed CD133+ & CD44+ population	[41]

*Salvia chinensis*	Stem	HCT116, COLO 205	10, 20, 40,60, 80, 100 mg/L	*Polyphenolic*	Terpenoids, phenolic acid, flavonoids, dibenzylcyclooctadiene	Apoptosis & loss of mitochondrial membrane	Induced G0/G1 cell cycle	[42]

*Rubus idaeus* L.	Fruit	HT-29, HT-115, Caco-2	3.125, 6.25, 12.5, 25, 50 mg/L	*Acetate*	Polyphenol, anthocyanin, ellagitannin	NM	Decreased population of cells in G1 phase	[47]
		LoVo	50 *μ*L	*Aqueous*	NM	Inhibited proliferation of LoVo	Suppression of the NF*κ*B pathway	[48]

*Curcuma longa*	Root	HT-29, HCT15, DLD1, HCT116	(i) Short-term assay: four 10-fold dilutions (100 to 0.1 mg/L) (ii) Long-term assay: 5, 10, 20 mg/L	*Ethanolic*	Curcumin (diferuloylmethane)	Inhibited formation of HCT116 spheroids	NM	[49]

*Eleutherococcus senticosus*	Root	HCT116	12.5, 25, 50, 100	*Methanolic*	Eleutherosides, triterpenoid saponins, glycans	NM	Activation of natural killer cells and thus enhancement of immune function	[50]

*Tabernaemontana divaricata* L.	Leaf	HT-29, HCT15	10, 30, 100 mg/L	*Ethyl acetate, chloroform, methanolic*	Alkaloids	NM	Inhibited the unwinding of supercoiled DNA	[45]

*Millingtonia hortensis*	Root, flower, leaf	RKO	50, 100, 200, 400, 800 mg/L	*Aqueous*	Phenylethanoid glycoside, squalene, salidroside, 2-phenyl rutinoside	Apoptosis induction	(i) Increase of fragmented DNA (ii) Decrease of the expression of antiapoptotic proteins, Bcl-xL and p-BAD	[46]
	Powder	RKO	200, 400, 800 *μ*g/mL	*Aqueous*	Water soluble compounds	Antiproliferative effect	NM	[51]

*Thai purple rice*	Seed	Caco-2, Cat. No. HTB-37	16.11 *μ*g/mL	*Methanol acidified*	Cyanidin-3-glucoside and peonidin-3-glucoside, anthocyanins, phenolic compounds	(i) Antioxidation of anthocyanins and phenols (ii) Antiproliferation of colon cancer cells	NM	[52]

*Annona muricata*	Leaf	HCT116, HT-29	11.43 ± 1.87 *μ*g/ml and 8.98 ± 1.24 *μ*g/ml	*Ethanolic*	Alkaloids, acetogenins, essential oils	Block of the migration and invasion of HT-29 and HCT116 cells	(i) Cell cycle arrest at G1 phase (ii) Disruption of MMP, cytochrome c leakage and activation	[53]
	NM	HT-29, HCT116	<4, <20 *μ*g/mL	*EtOAc*	Annopentocin A, annopentocin B, annopentocin C, cis- and trans-annomuricin-D-ones, annomuricin E	NM	Suppression of ATP production and NADH oxidase in cancer cells	[54]

*Pistacia lentiscus L.* var. *chia*	Leaf	HCT116	NM	*Ethanolic*	Resin, known as Chios mastic gum (CMG)	Causes several morphological changes typical of apoptosis in cell organelles	(i) Induction of cell cycle arrest at G1 phase (ii) Activation of pro-caspases 8, 9, 3	[55]
	Resin	HCT116	100 *μ*g/mL	*Hexane*	Caryophyllene	Induction of the anoikis form of apoptosis in human colon cancer HCT116 cells	(i) Induction of G1 phase arrest (ii) Loss of adhesion to the substrate	[56]

*American ginseng (Panax quinquefolius)*	Biological constituents	HCT116	0-2.0 mg/mL	*Aqueous*	Ginseng (GE) or its ginsenoside (GF) and polysaccharide (PS)	Proliferation was inhibited by GE, GF, and PS in wild-type and p21 cells	(i) Cells arrest in G0/G1 phase and increment of p53 and p21 proteins (ii) Increment of Bax and caspase 3 proteins expression	[57]

*Purple-fleshed potatoes*	Fruit	Colon cancer stem cells	5.0 *μ*g/mL	*Ethanol, methanol, ethyl acetate*	Anthocyanin, *β*-catenin, cytochrome c	Critical regulator of CSC proliferation and its downstream proteins (c-Myc and cyclin D1) and elevated Bax and cytochrome c	(i) Cytochrome c levels were elevated regardless of p53 status (ii) Mitochondria-mediated apoptotic pathway (iii) Suppressed levels of cytoplasmic and nuclear *β*-catenin	[58]

*Phaseolus vulgaris*	Leaf	HT-29	NM	*Ethanolic*	Polysaccharides, oligosaccharides	Changes in genes involved or linked to cell cycle arrest	(i) Inactivation of the retinoblastoma phosphoprotein (ii) Induction of G1 arrest (iii) Suppression of NF-jB1 (iv) Increase in EGR1 expression	[59]

*Opuntia spp.*	Fruit	HT-29	5.8 ± 1.0, 7.5 ± 2.0, 12 ± 1% (*V*/*V*)	*Hydroalcoholic*	Betacyanins, flavonoids (isorhamnetin derivatives) and phenolic acids (ferulic acid)	NM	Induced cell cycle arrest at different checkpoints—G1, G2/M, and S	[60]

*Suillus luteus*	NM	HCT15	400 *μ*g/mL	*Methanolic*	Protocatechuic acid, cinnamic acid, *α*-tocopherol, *β*-tocopherol, mannitol, trehalose, polyunsaturated fatty acids, monounsaturated fatty acids, saturated fatty acids	(i) Increase in the cellular levels of p-H2A.X, which is suggestive of DNA damage	(i) Inhibition of cell proliferation in G1 phase (ii) Increase in the cellular levels of p-H2A.X	[61]

*Poncirus trifoliata*	Leaf	HT-29	0.63 *μ*M	*Aqueous (in acetone)*	*β*-Sitosterol, 2-hydroxy-1,2,3-propanetricarboxylic acid 2-methyl ester	Arrest of cell growth was observed with *β*-sitosterol	NM	[62]

*Rosmarinus officinalis L.*	Leaf	SW 620, DLD-1	0-120 *μ*g/mL	*Methanolic*	Polyphenols	Antiproliferative effect of 5-FU	Downregulation of TYMS and TK1, enzymes related to 5-FU resistance	[63]
	Leaf	HT-29	SC-RE 30 *μ*g/mL and CA 12.5 *μ*g/mL	*Ethanolic*	Polyphenols (carnosic acid (CA) and carnosol)	(i) Upregulation of VLDLR gene as the principal contributor to the observed cholesterol accumulation in SC-RE-treated cells (ii) Downregulation of several genes involved in G1-S	Activation of Nrf2 transcription factor and common regulators, such as XBP1 (Xbp1) gene related to the unfolded protein response (UPR)	[64]
	NM	HT-29	10, 20, 30, 40, 50, 60, 70 *μ*g/mL	*NM*	Carnosic acid, carnosol, rosmarinic acid, rosmanol	NM	NM	[65]
	Leaf	HGUE-C-1, HT-29, and SW480	20–40 mg/mL	*CO2-supercritical fluid extract*	Carnosic acid, carnosol, and betulinic acid	NM	(i) Prooxidative capability by increasing the intracellular generation of ROS (ii) Activation of Nrf2	[66]

*Glehnia littoralis*	Leaf	HT-29	50 mg/mL	*Methanolic*	Bergapten, isoimpinellin, xanthotoxin, imperatorin, panaxydiol, falcarindiol, falcarinol	Induced apoptosis by the decreased expression of the antiapoptotic Bcl-2 mRNA	(i) Reduced expression of Bcl-2 (ii) Reduced expression levels of iNOS and COX-2	[67]

*Verbena officinalis*	Leaf	HCT116	20 mg/mL	*Aqueous*	Phenylethanoid glycosides, diacetyl-O-isoverbascoside, diacetyl-O-betonyoside A, and diacetyl-O-betonyoside A	(i) Substantial tumor cell growth inhibitory activity (ii) Time-dependent cytotoxicity against both cell lines	(i) Increased lipophilicity of molecules seemed to be responsible for enhanced cytotoxicity (ii) Antiproliferative activity is determined by the number of acetyl groups and also by their position in the aliphatic rings	[68]

*Mentha spicata*	Leaf	RCM-1	12.5 *μ*g/mL	*N-Hexane*	Acetic acid 3-methylthio propyl ester (AMTP), methyl thio propionic acid ethyl ester (MTPE)	Exhibited antimutagenic activity	Auraptene (7-geranyloxycoumarin) having a monoterpene moiety and *β*-cryptoxanthin (one of the tetraterpenes) increased antibody production	[69]

*Euphoria longana Lam.*	Seed	SW 480	0–100 *μ*g/mL	*Ethanolic*	Corilagin, gallic acid, ellagic acid	(i) Antiangiogenetic properties (ii) All fractions showed the anti-VEGF secretion activity	Release and expression of VEGF indicated that all fractions showed the anti-VEGF secretion activity	[70]

*Sutherlandia frutescens*	Flower	Caco-2	1/50 dilution of the ethanolic extract	*Ethanolic*	Amino acids, including L-arginine and L-canavanine, pinitol, flavonoids, and triterpenoid saponins as well as hexadecanoic acid and *γ*-sitosterol	Disruption of the key molecules in the PI3K pathway thereby inducing apoptosis	Decrease in cell viability and increment in pyknosis as well as loss in cellular membrane integrity	[71]

*Melissa officinalis*	Leaf	HT-29, T84	346, 120 *μ*g/mL	*Ethanolic*	Phenolic acids (rosmarinic acid, coumaric acid, caffeic acid, protocatechuic acid, ferulic acid, chlorogenic acid), flavonoids, sesquiterpenes, monoterpenes, triterpenes	(i) Inhibited proliferation of colon carcinoma cells (ii) Induced apoptosis through formation of ROS	(i) G2/M cell cycle arrest (ii) Cleavage of caspases 3 and 7 (iii) Induced phosphatidylserine externalization in colon carcinoma cells (iv) Induced formation of ROS in colon carcinoma cells	[72]

*Sargassum cristaefolium*	Leaf	HT-29	500 mg/mL	*Ethanolic*	Fucoidans	(i) Reduction of free radicals (ii) DPPH radical scavenging	Accumulation of cells in G0/G1 phase	[73]

*Hedyotis diffusa*	NM	HT-29	400 mg/mL	*Ethanolic and then DMSO*	Octadecyl (E)-p-coumarate, P-E-methoxy-cinnamic acid, ferulic acid, scopoletin, succinic acid, aurantiamide acetate, rubiadin	Suppress tumor cell growth and induce the apoptosis of human CRC cells	(i) Block G1/S progression (ii) Induce the activation of caspases 9 and 3 (iii) Inhibit IL-6-mediated STAT3 activation (iv) Downregulate the mRNA and protein expression levels of cyclin D1, CDK4, Bcl-1, and Bax	[74]

*Zingiber officinale Roscoe*	Peel	LoVo	100 mg/mL	*Ethanolic*	Linoleic acid methyl ester, *α*-zingiberene, and zingiberone	Interesting antiproliferative activity against colorectal carcinoma	NM	[75]

*Scutellaria barbata*	Leaf	LoVo	413.3 mg/L	*Methanolic*	Scutellarein, scutellarin, carthamidin, isocarthamidin, wogonin	Induce cell death in the human colon cancer cell line	Increase in the sub-G1 phase and inhibition of cell growth	[76]

*Pistacia atlantica, Pistacia lentiscus*	Resin	HCT116	100 *μ*g/mL	*Hexane extract*	Caryophyllene	Induce the anoikis form of apoptosis in human colon cancer HCT116 cells	(i) Induce G1 phase arrest (ii) Loss of adhesion to the substrate	[56]

*Citrus reticulata*	Peel	SNU-C4	100 *μ*g/mL	*Methanolic*	Limonene, geranial, neral, geranyl acetate, geraniol, *β*-caryophyllene, nerol, neryl acetate	Induce the apoptosis on SNU-C4, human colon cancer cells	Expression of proapoptotic gene, Bax, and major apoptotic gene, caspase 3	[77]

*Echinacea pallida, Echinacea angustifolia, Echinacea purpurea*	Root	COLO320	150 mg/mL	*Hexanic*	Caffeic acid derivatives, alkylamides, polyacetylenes, polysaccharides	Induce apoptosis and promote nuclear DNA fragmentation	(i) Induce apoptosis by increasing caspase 3/7 activity (ii) Promote nuclear DNA fragmentation	[78]

*Nasturtium officinale*	Leaf	HT-29	50 *μ*L/mL	*Methanolic*	Phenethyl isothiocyanate, 7-methylsulfinylheptyl, 8-methylsulfinyl	(i) Inhibition of initiation, proliferation, and metastasis	(i) Inhibited DNA damage (ii) Accumulation of cells in S phase of the cell cycle	[79]

*Polysiphonia*	NM	SW480, HCT15, HCT116, DLD-1	20 and 40 *μ*g/mL	*Methanolic*	2,5-Dibromo-3,4-dihydroxybenzyl n-propyl ether	Potentially could be used as a chemopreventive agent against colon cancer	(i) Inhibited Wnt/*β*-catenin pathway (ii) Repressed CRT in colon cancer cells (iii) Downregulated cyclin D1 (iv) Activated the NF*κ*B pathway	[80]

*Aristolochia debilis Sieb. et Zucc.*	Stem	HT-29	200 *μ*g/mL	*Methanolic*	Aristolochic acid, nitrophenanthrene carboxylic acids	Inhibition of proliferation and induction of apoptosis in HT-29 cells	(i) Induction of sub-G1 cell cycle (ii) Generation of ROS and decrease of the MMP (iii) Bax overexpression and increase of Bax/Bcl-2 ratio	[81]

*Myrtaceae*	Leaf	HCT116	100 *μ*g/mL (in vitro), 200 and 100 *μ*g/disc (*in vivo*)	*Methanolic*	Phenols, flavonoid, betulinic acid	Strong inhibition of microvessel outgrowth	(i) Inhibition of tube formation on Matrigel matrix (ii) Inhibition of HUVECS migration (*in vitro*) (iii) Decreased nutrient and oxygen supply	[82]

*Spica prunellae*	Leaf	HT-29	200 mg/mL (*in vitro*), 600 mg/mL (*in vivo*)	*Ethanolic*	Rosmarinic acid	Inhibits CRC cell growth	(i) Suppresses STAT3 phosphorylation (ii) Regulates the expression of Bcl-2, Bax, cyclin D1, CDK4, VEGF-A, and VEGFR-2	[83]

*Phytolacca americana*	Root	HCT116	3200 *μ*g/mL	*Ethanolic*	Jaligonic acids, kaempferol, quercetin, quercetin 3-glucoside, isoquercitrin, ferulic acid	Control of growth and spread of cancer cells	Reduction in the expressions of MYC, PLAU, and TEK	[84]

*Morus alba*	Leaf	HCT15	13.8 *μ*g/mL	*Methanolic*	Epicatechin, myricetin, quercetin hydrate, luteolin, kaempferol, ascorbic acid, gallic acid, pelargonidine, p-coumaric acid	Cytotoxic effect on human colon cancer cells (HCT15)	(i) Apoptosis induction also involved in the downregulation of iNOS (ii) Fragmentation of DNA (iii) Upregulation of caspase 3 activity	[85]

*Rhodiola imbricata*	Leaf	HT-29	200 *μ*g/mL	*Acetone and methanolic*	Phenols, tannins, and flavonoids	(i) Antioxidant activity (ii) Inhibited proliferation of HT-29 cells	(i) Scavenge free radicals (ii) DPPH radical scavenging activity (iii) Increased metal chelating activity	[86]

*Asiasarum heterotropoides F.*	Dried *A. radix*	HCT116	20 mg/mL	*Ethanolic*	Asarinin and xanthoxylol	Inhibition of the growth of HCT116 cells	(i) Caspase-dependent apoptosis (ii) Regulation of p53 expression at transcription level	[87]

*Podocarpus elatus*	Fruit	HT-29	500 mg/mL	*Methanolic*	Phenolic and anthocyanin	Reduction of proliferation of colon cancer cells	(i) Cell cycle delay in S phase (ii) 93% downregulation of telomerase activity and decrease in telomere length (iii) Induced morphological alterations to HT-29 cells	[88]

*Echinacea purpurea*	Flower	Caco-2, HCT116	0–2,000 mg/mL	*Hydroethanolic*	Cichoric acid	(i) Inhibition of proliferation (ii) Decreased telomerase activity in HCT116 cells	(i) Decreased telomerase activity (ii) Activation of caspase 9 (iii) Cleavage of PARP (iv) Downregulation of *β*-catenin	[89]
	Root	COLO320	150 mg/mL	*Hexanic*	Caffeic acid derivatives, alkylamides, polyacetylenes, polysaccharides	Induce apoptosis by increasing significantly caspase 3/7 activity and promote nuclear DNA fragmentation	(i) Increase significantly caspase 3/7 activity (ii) Promote nuclear DNA fragmentation	[78]

*Hop (Humulus lupulus L.), Franseria artemisioides*	Leaf	NM	100 mg/kg b.w./day	*Aqueous*	Coumarin, lignans, quinones	30% reduction of tumor-induced neovascularization	NM	[90]
	NM	Caco-2	NM	*Ethanolic*	Phenolic compounds, flavonoid, diterpenes	Digestive, gastroprotective, antiseptic, anti-inflammatory, and antiproliferative activity	NM	[91]
	Fruit	NL-17	0, 50, 100, 150 *μ*g/mL	*Methanolic*	*α*-Mangostin (xanthone)	NM	(i) Induction of caspase 3 and caspase 9 activation (ii) Induced cell cycle arrest at G1/G0 phase	[92]
	Stem, bark	HT-29	50 *μ*g/mL	*Chloroform-soluble*	*β*-Mangostin, garcinone D, cratoxyxanthone	Cytotoxic activity against HT-29 human colon cancer	Inhibition of p50 and p65 activation	[93]

*Annona squamosa Linn*	Leaf	HCT116	8.98 *μ*g/mL	*Crude, Aq ethyl acetate*	Acetogenins (annoreticuin & isoannoreticuin) and alkaloids dopamine, salsolinol, and coclaurine	Inhibition of growth and proliferation of tumor cells	(i) Reactive oxygen species (ROS) formation, lactate dehydrogenase (LDH) release (ii) Activation of caspases 3/7, 8, and 9	[94]

*Derris scandens*	Stem	HT-29	5-15 *μ*g/mL	*Ethanolic*	Benzyls and isoflavones (genistein, coumarins, scandinone)	Apoptosis and mitotic catastrophe of human colon cancer HT-29 cells	(i) Inhibition of *α*-glucosidase activity (ii) Scavenge free radicals	[95]

*Eupatorium cannabinum*	Aerial parts	HT-29	25 *μ*g/mL	*Ethanolic*	Pyrrolizidine alkaloids (senecionine, senkirkine, monocrotaline, echimidine)	Induced alteration of colony morphology	(i) Upregulation of p21 and downregulation of NCL, FOS, and AURKA (ii) Mitotic disruption and nonapoptotic cell death via upregulation of Bcl-xL, limited TUNEL labeling, and nuclear size increase	[96]

*Sorghum bicolor*	The dermal layer of stalk	HCT116 & colon cancer stem cells	>16 and 103 *μ*g/mL	*Phenolic-rich ethanolic, acetone*	Apigeninidin & luteolinidin	Antiproliferative	Target p53-dependent and p53-independent pathways	[97]
	Dermal and seed head	CCSC	NM	*Methanolic*	Apigeninidin, luteolinidin, malvidin 3-O-glucoside, apigenin, luteolin, naringenin, naringenin 7-O-glucoside, eriodictyol 5-glucoside, taxifolin, catechins	NM	(i) Elevation of caspase 3/7 activity (ii) Decrease in *β*-catenin, cyclin D1, c-Myc, and survivin protein levels (iii) Suppression of Wnt/*β*-catenin signaling in a p53-dependent (dermal layer) and partial p53-dependent (seed head) manner	[98]

*Hibiscus cannabinus*	Seed	HCT116	KSE (15.625 *μ*g/mL to 1,000 *μ*g/mL)	*Ethanolic*	Gallic acid, p-hydroxybenzoic acid, caffeic acid, vanillic acid, syringic acid, and p-coumaric and ferulic acids	Cytotoxic activity against human colon cancer HCT116 cells	Apoptosis via blockade of mid G1-late G1-S transition thereby causing G1 phase cell cycle arrest	[99]

*Salix aegyptiaca* L.	Bark	HCT116 & HT-29	300 *μ*g/mL	*Ethanolic*	Catechin, salicin, catechol and smaller amounts of gallic acid, epigallocatechin gallate (EGCG), quercetin, coumaric acid, rutin, syringic acid, and vanillin	Anticarcinogenic effects in colon cancer cells	Apoptosis via inhibition of phosphatidylinositol 3-kinase/protein kinase B and mitogen-activated protein kinase signaling pathways	[100]

*Rubus coreanum*	Fruit	HT-29	400 *μ*g/mL	*Aqueous*	Polyphenols, gallic acid, sanguine	Induction of apoptosis	(i) Induced activity of caspases 3, 7, and 9 (ii) Cleavage of poly(adenosine diphosphate-ribose) polymerase	[101]

*Codonopsis lanceolata*	Root	HT-29	200 *μ*g/mL	*N-Butanol fraction*	Tannins, saponins, polyphenolics, alkaloids	Apoptosis in human colon tumor HT-29 cells	(i) Induced G0/G1 arrest (ii) Enhancement of expression of caspase 3 and p53 and of the Bax/Bcl-2 ratio	[102]

*Gleditsia sinensis*	Thorn	HCT116	800 *μ*g/mL	*Aqueous*	Flavonoid, lupine acid, ellagic acid glycosides	(i) Increase in p53 levels (ii) Downregulation of the checkpoint proteins, cyclin B1, Cdc2, and Cdc25c	Inhibition of proliferation of colon cancer cells	[90]
	Thorn	HCT116	600 *μ*g/mL	*Ethanolic*	NM	Inhibitory effect on proliferation of human colon cancer HCT116 cells	(i) Caused cell cycle arrest at G2/M phase together with a decrease of cyclin B1 and Cdc2 (ii) Progression from G2/M phase	[91]

*Ligustrum lucidum*	Fruit	DLD-1	50 *μ*g/mL	*Aqueous*	Oleanolic acid, ursolic acid	Inhibited proliferation	(i) Reduction of Tbx3 rescued the dysregulated P14ARF-P53 signaling	[94]

*Zingiber officinale*	Rhizome	HCT116	5 *μ*M	*Ethanolic*	6-Paradol, 6- and 10-dehydrogingerdione, 6- and 10-gingerdione, 4-, 6-, 8-, and 10-gingerdiol, 6-methylgingerdiol, zingerone, 6-hydroxyshogaol, 6-, 8-, 10-dehydroshogaol, diarylheptanoids	Inhibitory effects on the proliferation of human colon cancer cells	(i) Arrest at G0/G1 phase (ii) Reduced DNA synthesis	[103]

*Grifola frondosa*	Fruit	HT-29	10 ng/mL	*Aqueous*	Phenolic compounds (pyrogallol, caffeic acid, myricetin, protocatechuic acid)	Inhibition of TNBS-induced rat colitis	Induced cell cycle progression in G0/G1 phase	[104]

*Cucumaria frondosa*	The enzymatically hydrolyzed epithelium of the edible	HCT116	<150 *μ*g/mL	*Hydroalcoholic*	Monosulphated triterpenoid glycoside frondoside A, the disulphated glycoside frondoside B, the trisulphated glycoside frondoside C	Inhibition of human colon cancer cell growth	(i) Inhibition at S and G2-M phases with a decrease in Cdc25c and increase in p21WAF1/CIP (ii) Apoptosis associated with H2AX phosphorylation and caspase 2	[105]

*Rolandra fruticosa*	Leaf & twigs	HT-29	10 and 5 mg/kg/day	*Methanolic*	Sesquiterpene lactone (13-acetoxyrolandrolide)	Antiproliferative effect against human colon cancer cells	Inhibition of the NF*κ*B pathway, NF*κ*B subunit p65 (RelA), upstream mediators IKK*β* and oncogenic K-ras	[106]

*Cydonia oblonga Miller*	Leaf & Fruit	Caco-2	250–500 *μ*g/mL	*Methanolic*	Phenolic compound (flavonol and flavone heterosides, 5-O-caffeoylquinic acid)	Antiproliferative effect against human kidney and colon cancer cells	(i) Suppression of factor activation, nuclear factor-kB (NF*κ*B) activation, protein-1 (AP-1) transcription factor, mitogen protein kinases (MAPKs), protein kinases (PKs), namely, PKC, growth-factor receptor- (GFR-) mediated pathways and angiogenesis (ii) Cell cycle arrest and induction of apoptosis, antioxidant, and anti-inflammatory effects	[107]

*Morchella esculenta*	Fruits	HT-29	820 mg/mL	*Methylene chloride*	Steroids (mainly ergosterol derivatives) & polysaccharides & galactomannan	Antioxidant activity in HT-29 colon cancer cells	Inhibition of NF-B activation in the NF-B assay	[108]

*Sedum kamtschaticum*	Aerial part	HT-29	0–0.5 mg/mL	*Methanolic*	Buddlejasaponin IV	Induced apoptosis in HT-29 human colon cancer cells	Induction of apoptosis via mitochondrial pathway by downregulation of Bcl-2 protein levels, caspase 3 activation, and subsequent PARP cleavage	[109]

*Ginseng and Glycyrrhiza glabra*	Leaf	HT-29	500 *μ*L	*Aqueous*	Uracil, adenine, adenosine, Li-glycyrrhetinic acid, quiritin	NM	Antiproliferative effect determination of the protein levels of p21, cyclin D1, PCNA, and cdk-2, which are the key regulators for cell cycle progression	[110]

*Orostachys japonicus*	Leaf & stem	HT-29	2 mg/mL	*Aqueous*	Flavonoids, triterpenoids, 4-hydroxybenzoic acid, 3,4-dihydroxybenzoic acid, polysaccharide	Antiproliferation in HT-29 colon cancer cells	Inhibited proliferation at G2 point of the cell cycle and apoptosis via tumor suppressor protein p53	[111]

*Ginkgo biloba*	Fruit & leaf	HT-29	20–320mg/L	*Aqueous*	Terpene lactones and flavonoid glycosides	(i) Inhibited progression of human colon cancer cells (ii) Induced HT-29 cell apoptosis	Increase in caspase 3 activities and elevation in p53 MRN reduction in Bcl-2 mRNA	[112]

*Oryza sativa*	Seed	HT-29, SW 480, HCEC	100 *μ*g/mL	*Ethyl acetate*	Phenolic compound (tricin, ferulic acid, caffeic acid, and methoxycinnamic acid)	Inhibition of the human colon cancer cell growth	(i) Induced apoptosis by enhanced activation of caspases 8 and 3 (ii) Decrease of the number of viable SW480 and HCEC cells (iii) Reduced colony-forming ability of these cells	[113]

*Cnidium officinale Makino*	Root	HT-29	305.024/mL	*Ethanolic*	Osthole, auraptenol, imperatorin	Inhibited proliferation of human colon cancer cells (HT-29)	Inhibition of the cellular proliferation via G0/G1 phase arrest of the cell cycle and induced apoptosis	[114]

*Cnidium officinale Makino*	Root	HT-29	0.1-5 mg/mL	*Aqueous*	N-(3-(Aminomethyl)benzyl)acetamidine	Inhibited the invasiveness of cytokine-treated HT-29 cells through the Matrigel-coated membrane in a concentration-dependent manner	(i) Reduction of HT-29 cell invasion through the Matrigel (ii) Inhibited cytokine-mediated NO production, iNOS expression, and invasiveness of HT-29 cells (iii) Inhibited MMP-2 activity	[115]

*Long pepper (PLX)*	Fruit	HT-29 and HCT116	0.10 mg/mL	*Ethanolic*	Piperidine alkaloids, piperamides, piperlongumine	(i) Induction of apoptosis, following DNA fragmentation in HT-29 colon cancer cells in a time-dependent manner (ii) Induced caspase-independent apoptosis	Induced whole cell ROS production	[116]

*Achyranthes aspera*	Root	COLO 205	50-100 and 150-200 *μ*g/mL	*Ethanolic (EAA) and aqueous (AAA) root extracts* *Aqueous*	Phenolic compounds	(i) Enhanced growth inhibitory effects of AAA towards COLO 205 cells in contrast to EAA (ii) Stimulatory role of AAA in the activation of cell cycle inhibitors	(i) Triggered mitochondrial apoptosis pathway and S phase cell cycle arrest (ii) Increased levels of caspase 9, caspase 3, and caspase 3/7 activity	[117]

*Thymus vulgaris*	Leaf	HCT116	0.2, 0.4, 0.6, 0.8 mg/mL		Carvacrol and thymol	Inhibited proliferation, adhesion, migration, and invasion of cancer cells		[118]

*Dictyopteris undulata*	NM	SW480	40 *μ*g/mL	*Ethanolic*	Cyclozonarone benzoquinone	NM	Induced apoptosis by reducing Bcl-2 levels, upregulating Bax, and disrupting the mitochondrial membrane potential, leading to the activation of caspases 3 and 9	[119]

*Dendrobium microspermae*	NM	HCT116	0.25, 0.5, 1.0 mg/mL	*Methanolic*	NM	NM	Upregulation of Bax and caspases 9 and 3 and downregulation of Bcl-2 expression of genes	[120]

*Cannabis sativa*	Dry flower & leaf	DLD-1 and HCT116	0.3–5 *μ*M	*Methanolic*	Cannabidiol, phytocannabinoids	Reduced cell proliferation in a CB1-sensitive	(i) Reduced AOM-induced preneoplastic lesions and polyps (ii) Inhibited colorectal cancer cell proliferation via CB1 and CB2 receptor activation	[121]

*Phoenix dactylifera* L.	Fruit	Caco-2	0.2 mg/mL	*Aqueous*	Phenolic acids (gallic, protocatechuic, hydroxybenzoic, vanillic, isovanillic, syringic, caffeic, ferulic, sinapic, p-coumaric, isoferulic), flavonoid glycosides (quercetin, luteolin, apigenin, and kaempferol), and anthocyanidins	Increasing beneficial bacterial growth and inhibition of proliferation of colon cancer cells	NM	[122]

*Melia toosendan*	Fruit	SW480, CT26	0, 10, 20, 30, 40, 50 *μ*g/mL	*Ethanolic*	Triterpenoids, flavonoids, polysaccharide, limonoids	NM	(i) Inhibited cell proliferation of SW480 and CT26 by promoting apoptosis as indicated by nuclear chromatin condensation and DNA fragmentation (ii) Induced caspase 9 activity which further activated caspase 3 and poly(ADP-ribose) polymerase cleavage, leading the tumor cells to apoptosis	[123]

*Crocus sativus* L.	Flower	HCT116	0.25, 0.5, 1, 2, 4 *μ*g/mL	*Ethanolic*	Carotenoid, pigment, crocin, crocetin	Induced DNA damage and apoptosis	(i) Induction of a p53 pattern-dependent caspase 3 activation with a full G2/M stop (ii) Induced remarkable delay in S/G2 phase transit with entry into mitosis	[124]
	Tepals and leaf	Caco-2	0.42 mg/mL	*NM*	Polyphenols, glycosides of kaempferol, luteolin, and quercetin	Proliferation of Caco-2 cells was greatly inhibited	NM	[125]

*Luffa echinata*	Fruit	HT-29	50, 100, and 200 *μ*g/mL	*Methanolic*	Amariin, echinatin, saponins, hentriacontane, gypsogenin, cucurbitacin B, datiscacin, 2-*O*-*β*-D-glucopyranosyl cucurbitacin B, and 2-*O*-*β*-D-glucopyranosyl cucurbitacin S	Increase in the population of apoptotic cells	(i) Inhibited the cellular proliferation of HT-29 cells via G2/M phase arrest of the cell cycle (ii) Induced apoptotic cell death via ROS generation (iii) Accumulation of caspase 3 transcripts of HT-29 cells	[126]

*Vitis aestivalis hybrid*	Fruits (wine)	CCD-18Co	25, 50, 100 *μ*g/mL	NM	Polyphenolics	NM	(i) Decreased mRNA expression of lipopolysaccharide- (LPS-) induced inflammatory mediators NF*κ*B, ICAM-1, VCAM-1, and PECAM-1 (ii) Enhanced expression of miR-126 (iii) Decreased gene expression and reduced activation of the NF*κ*B transcription factor, NF*κ*B-dependent (iv) Decrease in ROS 113MAH	[127]

*Xylopia aethiopica*	Dried fruit	HCT116	0, 5, 10, 15, 20, 25, 30 *μ*g/mL	*Ethanolic*	Ent-15-oxokaur-16-en-19-oic acid (EOKA)	NM	(i) Induced DNA damage, cell cycle arrest in G1 phase, and apoptotic cell death	[128]

*Sorghum*	Grain	ER-*β*; nonmalignant young adult mouse colonocytes	1, 5, 10, 100 *μ*g/mL	*Aqueous*	Flavones (luteolin and apigenin), 3-deoxyanthocyanins naringenin (eriodictyol and naringenin)	Reduced cell growth via apoptosis	Increased caspase 3 activity	[129]
	NM	HT-29, HCT116	0.9-2.0 mg/mL	*Hydroethanolic*	Procyanidin B1, delphinidin-3-O-glucoside, tannin, cyanidin-3-O glucoside	(i) Significantly arrested HT-29 cells in G1 (ii) Highest growth inhibition (iii) Increased percentage of apoptotic cells	(i) Downregulation of apoptotic proteins, such as cIAP-2, livin, survivin, and XIAP, was seen in HCT116 cells (ii) Inhibition of tyrosine kinase	[130]

*Panax notoginseng (Burk.) F.H. Chen*	Root	LoVo and Caco-2	0, 100, 250, and 500 *μ*g/mL	*Alcoholic*	Saponin, ginsenoside	NM	Delay in progression of the G0/G1, S, or G2/M cell cycle phases	[131]

*Brassica oleracea* L. var. *italica*	Broccoli florets	HCT116	0, 1, 2.5, 5, 10 *μ*g/mL	*Ethanolic*	Glucoiberin, 3 hydroxy,4(*α*-L-rhamnopyranosyloxy), benzyl glucosinolate 4-vinyl-3-pyrazolidinone 4-(methyl sulphinyl), butyl thiourea, *β*-thioglucoside N-hydroxysulphates	NM	NM	[132]

*Cistanche deserticola*	Dried stem	SW480	*In vivo:* 0.4 g/kg/day *In vitro:* 100 *μ*g/mL	*Aqueous*	Polysaccharides, phenylethanoid glycosides	(i) Decreased number of mucosal hyperplasia and intestinal helicobacter infection (ii) Increased number of splenic macrophage, NK cells, and splenic macrophages	Decreased frequency of hyperplasia and *Helicobacter hepaticus* infection of the intestine	[133]

*Chaenomeles japonica*	Fruit	Caco-2 and HT-29	10, 25, 50, 75, 100, 125, 150 *μ*M CE	NM	Procyanidins	NM	NM	[134]

*Prunus mume*	Fruit	SW480, COLO, and WiDr	150, 300, and 600 *μ*g/mL	*Hydrophobic*	Triterpenoid saponins	NM	(i) Inhibited growth and lysed SW480, COLO, and WiDr (ii) Induction of massive cytoplasmic vacuoles	[135]

*Solanum lyratum*	NM	COLO 205	50, 100, 200, 300, 400 *μ*g/mL	*EtOH*	*β*-Lycotetraosyl	Induced S phase arrest and apoptosis	(i) Induced DNA fragments (ii) Increased the levels of p27, p53, cyclin B1, active-caspase 3, and Bax (iii) Decreased the levels of Cdk1, pro-caspase 9, Bcl-2 and NF-ÎB, p65, and p50	[136]

*Onopordum cynarocephalum*	Aerial parts	HCT116, HT-29	0, 0.04, 0.12, 0.2, 0.4, 1.2 mg/mL 0, 0.2, 0.4, 1.2, 2.0, 3.0 mg/mL	*Aqueous*	Flavonoids, lignans, and sesquiterpene lactones	NM	(i) Increase in the expression of proapoptotic proteins such as p53, p21, and Bax (ii) Inhibition of the antiapoptotic protein Bcl-2 (iii) Decrease in cyclin D1 protein	[137]

*Eleutherine palmifolia*	Bulbs	SW480	2.5, 5, 10 *μ*g/mL	*MeOH*	Eleutherin, isoeleutherin	NM	(i) Inhibited the transcription of TCF/*β*-catenin (ii) Decrease in the level of nuclear *β*-catenin protein	[138]

*Asparagus officinalis*	Spears	HCT116	76 *μ*g/mL	*Acetone*	Steroidal saponins (HTSA-1, HTSAP-2, HTSAP-12, HTSAP-6, HTSAP-8)	NM	(i) Inhibition of Akt, p70S6K, and ERK phosphorylation (ii) Induction of caspase 3 activity, PARP-1 cleavage, DNA fragmentation, G0/G1 cell cycle arrest by reducing the expression of cyclins D, A, and E	[139]

*Phyllanthus emblica L.*	Seed, pulp	HCCSCs, HCT116	200 *μ*g/mL	*Methanolic*	Trigonelline, naringin, kaempferol, embinin, catechin, isorhamnetin, quercetin	(i) Suppressed proliferation (ii) Induced apoptosis independent from p53 stemness property (in HCCSCs) (iii) Antiproliferative properties	(i) Suppressed cell proliferation and expression of c-Myc and cyclin D1 (ii) Induced intrinsic mitochondrial apoptotic signaling pathway	[140]

*Red grape*	NM	HT-29, HCT116	0.9-2.0 mg/mL	*Hydroethanolic*	Delphinidin glycosides, quercetin derivatives, delphinidin-3-O-glucoside (high), cyanidin-3-O-glucoside	(i) Highest growth inhibition (ii) Increased the percentage of apoptotic cells	(i) Downregulation of apoptotic proteins, such as cIAP-2, livin, survivin, and XIAP (ii) Inhibition of tyrosine kinase	[130]

*Black lentil*	NM	HT-29, HCT116	0.9-2.0 mg/mL	*Hydroethanolic*	Delphinidin glycosides, procyanidin B1, delphinidin-3-O-glucoside (high), cyanidin-3-O-glucoside	(i) Significantly arrested HT-29 cells in G1 (ii) Highest growth inhibition (iii) Increased percentage of apoptotic cells	(i) Downregulation of apoptotic proteins, such as cIAP-2, livin, survivin, and XIAP (ii) Inhibition of tyrosine kinase	[130]

*Graptopetalum paraguayense*	Leaf	Caco-2, BV-2	0.2, 0.4, 0.6, 0.8, 1.0 mg/mL	*Hydroethanolic*	Oxalic acid, hydroxybutanedioic acid, gallic acid, quercetin, chlorogenic acid glucans with fucose, xylose, ribose (GW100) arabino-rhamnogalactans (GW100E)	(i) Great potential in antiproliferation (ii) Significant immunomodulatory activities on BV-2 cells and interleukin-6 (IL-6) (GW100)	(i) Scavenging *α*, *α*-diphenyl-*β*-picrylhydrazyl radicals (DPPH) (GW100E excelled in scavenging DPPH), 2,2-azino-bis [3-ethylbenzothiazoline-6-sulfonic acid] radicals (ABTS), superoxide anions (O2) (GW100) (ii) Significant inhibition of tumor necrosis factor-a (TNF-a), scavenging ABTS and O2	[141]

*Butea monosperma*	Flower	SW480	200, 370 *μ*g/mL	*Floral*	n-Butanol	Significant antiproliferative effect	(i) Significantly downregulated the expression of Wnt signaling proteins such as *β*-catenin, APC, GSK-3*β*, cyclin D1, and c-Myc (ii) Increased intracellular level of ROS	[142]

*Rehmannia glutinosa*	NM	CT26	5, 20, 80 *μ*M	*NM*	Catalpol	Inhibited proliferation and growth invasion of colon cancer cells	(i) Downregulated MMP-2 and MMP-9 protein expressions (ii) Reduction in the angiogenic markers secretions	[143]

*Telectadium dongnaiense*	Bark	HCT116	1.5, 2.0 *μ*g/mL	*MeOH extract*	4-Dicaffeoylquinic acid, quercetin 3-rutinoside, periplocin	NM	(i) Inhibition of *β-*catenin/TCF transcriptional activity and effects on Wnt/*β*-catenin (ii) Downregulation of the expression of Wnt target genes	[144]

*Gloriosa superba*	Root	SW620	30 ng/mL	*Protein hydrolysate extract*	Protein hydrolysate	NM	(i) Upregulation of p53 (ii) Downregulation of NF*κ*B	[145]

*Boswellia serrata*	Resin	HT-29	100, 150 *μ*g	*Methanolic*	Boswellic acid	Decreased cell viability	(i) Reduction in mPGES-1, VEGF, CXCR4, MMP-2, MMP-9, HIF-1, PGE2 expression (ii) Increment in the caspase 3 activity (iii) Inhibition of cell migration and vascular sprout formation	[146]

*Typhonium flagelliforme*	Leaf	WiDr	70 *μ*g/mL	*Ethyl acetate*	Glycoside flavonoid, isovitexin, alkaloids	NM	Inhibition of COX-2 expression	[28]

*Diospyros kaki*	Fruit	HT-29	2,000 *μ*g/mL	*Hydroacetone extract*	Polyphenol	Impaired cell proliferation and invasion	NM	[147]

*Carpobrotus edulis*	Leaf	HCT116	1,000 mg/mL	*Hydroethanolic*	Gallic acid, quercetin, sinapic acid, ferulic acid, luteolin 7-o-glucoside, hyperoside, isoquercitrin, ellagic acid, isorhamnetin 3-O-rutinoside	Inhibited proliferation	(i) Possession of high DPPH scavenging activity and effective capacity for iron binding (ii) Inhibition of NO radical, linoleic acid peroxidation, protein glycation, and oxidative damage	[148]

*Piper methysticum*	Root	HT-29	10, 20, 30, 40, 50 *μ*g/mL	*Aqueous*	11-Hydroxy-12-methoxydihydrokavain, 11-hydroxy-12-methoxydihydrokavain, prenyl caffeate, pinostrobin chalcone, 11-methoxytetrahydroyangonin, awaine, methysticin, dihydromethysticin, 5,6,7,8-tetrahydroyangonin, kavain, 7,8-dihydrokavain, yangonin, desmethoxyyangonin, flavokawain B	Inhibited the growth	NM	[26]

*Salvia ballotiflora*	Ground aerial parts	CT26	6.76 *μ*g/mL	*Hexane-washed chloroform extract*	19-Deoxyicetexone, 7,20-dihydroanastomosine, icetexone, 19-deoxyisoicetexone	Cytotoxic activity	NM	[149]

*Tinospora cordifolia*	Stem	HCT116	1, 10, 30, 50 *μ*M	*Hydroalcoholic*	Clerodane furano diterpene glycoside, cordifoliosides A and Β, sitosterol, ecdysterone, 2*β*,3*β*:15,16-diepoxy-4*α*, 6*β*-dihydroxy-13(16),14-clerodadiene-17,12:18,1-diolide	Induced chromatin condensation and fragmentation of nuclei of few cells	(i) Considerable loss of MMP (ii) Decreased in mitochondria function (iii) Increased cytochrome c in the cytosol (iv) Induced ROS/oxidative stress (v) Increased autophagy	[150]

*Euterpe oleracea*	Fruit	NM	35 *μ*g/mL	*Hydroethanolic*	Vanillic acid, orientin, isoorientin	NM	(i) Scavenging capacity towards ROO and HOCl (ii) Inhibition of nitroso compound formation	[151]

*Salvia miltiorrhiza*	NM	HCT116	7.4 ± 1.0, 4.4 ± 0.5 *μ*g/mL	*Ethanolic*	Diterpene quinone	NM	Decreased levels of pro-caspases 3 and 9	[152]

*Coffea*	Bean	HCT116	1 mg/mL	*Aqueous*	Chlorogenic acid complex (CGA7)	NM	(i) DNA fragmentation, PARP-1 cleavage, caspase 9 activation, downregulation of Bcl-2 and upregulation of Bax	[153]

*Illicium verum*	Fruit	HCT116	10 mg/mL	*Ethanolic*	Gallic acid quercetin	Induction of apoptosis and inhibition of key steps of metastasis	NM	[154]

*Garcinia propinqua Craib*	Leaf	HCT116	NM	*CH2Cl2 extract*	Benzophenones, xanthones, and caged xanthones	Potent inhibitory cytotoxicities	NM	[155]
	Stem, bark	HCT116	14.23, 23.95 *μ*M	*MeOH, CH_2_Cl_2_, and EtOAc extract*	Xerophenone A, doitunggarcinones A and B, sampsonione, 7*β*-H-11-benzoyl-5*α*-ydroxy-6, 10-tetramethyl-1-(3-methyl-2-butenyl)-tetracyclotetradecane-2,12,14-trione, hypersampsone M, assiguxanthone A (cudraxanthone Q), 40 10-O-methylmacluraxanthone (16), 41- and 5-O-methylxanthone V1	NM	NM	[156]

*Malus pumila Miller cv. Annurca*	Fruit	Caco-2	400 mg/L	*Methanolic*	Chlorogenic acid, (+)catechin, (–)epicatechin, isoquercetin, rutin, phloridzin, procyanidin B2, phloretin, quercetin	WNT inhibitors and reduced WNT activity elicited by WNT5A	NM	[157]

*Malus domestica cv. Limoncella*	Fruit	Caco-2	400 mg/L	*Methanolic*	Chlorogenic acid, (+)catechin, (–)epicatechin, isoquercetin, rutin, phloridzin, procyanidin B2, phloretin, quercetin	WNT inhibitors and reduced WNT activity elicited by WNT5A	NM	[157]

*Coix lacryma-jobi* var. *ma-yuen*	Leaf	HCT116	0.5, 1 mg/mL	*Aqueous*	Coixspirolactam A, coixspirolactam B, coixspirolactam C, coixlactam, methyl dioxindole-3-acetate	NM	Inhibited migration, invasion, and adhesion via repression of the ERK1/2 and Akt pathways under hypoxic conditions	[158]

*Mesua ferrea*	Stem, bark	HCT116, HT-29	3.3, 6.6, and 11.8 *μ*g/mL	*NM*	Fractions (*α*-amyrin, SF-3, n-Hex)	Downregulation of multiple tumor promoter	Upregulation of p53, Myc/Max, and TGF-*β* signaling pathways	[159]

*Taraxacum*	Root	SGC7901, BGC823	3 mg/mL	*Aqueous*	NM	NM	Proliferation and migration through targeting lncRNA-CCAT1	[160]

*Portulaca oleracea*	Leaf	HT-29 CSCs	2.25 *μ*g/mL	*Alcoholic*	Oxalic, malic acid	NM	Inhibited expression of the Notch1 and *β*-catenin genes, regulatory and target genes that mediate the Notch signal transduction pathway	[161]

*Hordeum vulgare L.*	NM	HT-29	NM	*Aqueous & juice*	Protein, dietary fiber, the B vitamins, niacin, vitamin B6, manganese, phosphorus, carbohydrates	(i) Inhibited proliferation of cancer cells (ii) Cytotoxic activity	Free radical scavenging activity	[162]

*Paraconiothyrium sp.*	NM	COLO 205 and KM12	12.5 *μ*M	*Methyl ethyl ketone extract*	n-Hexane, CH_2_Cl_2_, EtOAc, and MeOH fractions (A−D)	(i) Growth inhibitory activity (ii) Antiproliferative effect	NM	[163]

*Mentha×piperita*	Leaf	HCT116	5, 10, 20, 30, 40, 50 *μ*g/mL	*Aqueous*	Polyphenols	NM	Inhibited replication of DNA and transcription of RNA which induce the ROS	[164]

*Mammea longifolia Planch. and Triana*	Fruit	SW480	25, 50, 100 *μ*g/mL	*Methanolic*	NM	NM	Mitochondria-related apoptosis and activation of p53	[165]

*Rollinia mucosa (Jacq.) Baill.*	NM	HCT116, SW-480	<4, <20 *μ*g/mL	*EtOH*	Rollitacin, jimenezin, membranacin, desacetyluvaricin, laherradurin	Cytotoxic activity	NM	[54]

*Annona diversifolia Saff.*	NM	SW-480	0.5 *μ*g/mL	NM	Cherimolin-2	Cytotoxic activity	NM	[54]

*A. purpurea Moc. & Sessé ex Dunal*	NM	HT-29	1.47 *μ*g/mL	*CHCl_3_-MeOH*	Purpurediolin, purpurenin, annoglaucin, annonacin A	Cytotoxic activity	NM	[54]

*Viguiera decurrens (A.Gray) A. Gray*	NM	NM	3.6 *μ*g/mL	*Hex; EtOAc; MeOH*	*β*-Sitosterol-3-O-*β*-D-glucopyranoside; *β*-D-glucopyranosyl oleanolate; *β*-sitosterol-3-O-*β*-D-glucopyranoside, and oleanolic acid-3-O-methyl-*β*-D-glucuronopyranoside ronoate	Cytotoxic activity	NM	[54]

*Helianthella quinquenervis (Hook.) A. Gray*	NM	HT-29	2-10 *μ*g/mL	NM	Demethylencecalin	Cytotoxic activity	NM	[54]

*Smallanthus maculatus (Cav.) H. Rob.*	NM	HCT15	<20 *μ*g/mL	*Acetone*	Fraction F-4, fraction F-5, ursolic acid	Cytotoxic activity	NM	[54]

*Bursera fagaroides (Kunth) Engl.*	NM	HF6	1.8×10^−4^ to 2.80 *μ*g/mL	*Hydroalcoholic*	Podophyllotoxin, *β*-peltatin-A methyl ether, 5′-desmethoxy-*β*-peltatin-A methyl ether, desmethoxy-yatein, deoxypodophyllotoxin, burseranin, acetyl podophyllotoxin	NM	(i) Inhibitor of microtubules (ii) Ability to arrest cell cycle in metaphase	[54]

*Viburnum jucundum C.V. Morton*	NM	HCT15	<20 *μ*g/mL	*Acetone*	Ursolic acid	Cytotoxic activity	NM	[54]

*Hemiangium excelsum (Kunth) A.C.Sm.*	NM	HCT15	<10 (*μ*g/mL)	*MeOH*	*PE, EtOAc, MeOH*	Cytotoxic activity	NM	[54]

*Hyptis pectinata (L.) Poit.*	NM	Col2	<4, <20 *μ*g/mL	NM	Pectinolide A, pectinolide B, pectinolide C, *α*-pyrone, boronolide, deacetylepiol-guine	Cytotoxic activity	NM	[54]

*H. verticillata Jacq.*	NM	Col2	<4,<20 *μ*g/mL	NM	Dehydro-*β*-peltatin, methyl ether dibenzylbutyrolactone, (-)-yatein, 4′-demethyl-deoxypodophyllotoxin	Nonspecific cytotoxic activity	NM	[54]

*H. suaveolens (L.)*	NM	HF6	2.8-12 *μ*g/mL	*Chloroform and butanol*	*β*-Apopicropodophyllin	Nonspecific cytotoxic activity	NM	[54]

*Salvia leucantha Cav.*	Leaf, root, stem	HF6, HT-29, HCT15	14.9, 12.7, 9.9 *μ*g/mL	*CHCl_3_*	NM	Cytotoxic activity	NM	[54]

*Vitex trifolia L.*	NM	HCT15	3.5 to <1 (*μ*g/mL)	*Hexane and dichloromethane*	Salvileucalin B, Hex: leaf, Hex: stem, DCM: leaf, DCM: stem	Cytotoxic activity	NM	[54]

*Persea americana Mill.*	NM	HT-29	<4 *μ*g/mL and <20 *μ*g/mL	*Ethanolic*	1,2,4-trihydroxynonadecan, 1,2,4-trihydroxyheptadec-16-ene, 1,2,4-trihydroxyheptadec-16-yne	Cytotoxic activity	NM	[54]

*Linum scabrellum*	Roots, aerial parts	HF6	0.2, 0.5, 2.3 *μ*g/mL	*Chloroform and butanol*	DCM: MeOH, 6MPTOXPTOX	NM	(i) Induction of cell cycle arrest in G2/M (ii) Inhibition of tubulin polymerization	[54]

*Phoradendron reichenbachianum (Seem.) Oliv.*	NM	HCT15	3.6, 3.9, and 4.3 *μ*g/mL	NM	Moronic acid	Cytotoxic activity	NM	[54]

*Cuphea aequipetala Cav.*	NM	HCT15	18.70 *μ*g/mL	*Acetone*	NM	Cytotoxic inactivity	NM	[54]

*Galphimia glauca Cav.*	NM	HCT15	0.63, 0.50, 1.99 *μ*g/mL	*EtOH, MeOH, aqueous*	NM	Cytotoxic activity	NM	[54]

*Mimulus glabratus Kunth*	NM	HF6	12.64 *μ*g/mL	*MeOH*	NM	Cytotoxic activity	NM	[54]

*Picramnia antidesma Sw.*	NM	HCT15	0.6 to 4.5 *μ*M	NM	10-Epi-uveoside, uveoside, picramnioside E, picramnioside D	Cytotoxic activity	NM	[54]

*Penstemon barbatus (Cav.) Roth*	NM	HF6	15.19 *μ*g/mL	*MeOH*	NM	Cytotoxic activity	NM	[54]

*P. campanulatus (Cav.) Willd.*	NM	HF6	6.74 *μ*g/mL	*MeOH*	NM	Cytotoxic activity	NM	[54]

*Veronica americana Schwein. ex Benth.*	NM	HF6	0.169 and 1.46 *μ*g/mL	*MeOH*	NM	Cytotoxic activity	NM	[54]

*Zea mays* L.	NM	HCT116, SW-480, SW-620	NM	NM	13-Hydroxy-10-oxo-trans-11-octadecenoic acid	Cytotoxic activity	NM	[54]

*Colubrina macrocarpa (Cav.) G. Don*	NM	HCT15	10, 2.1, 9.1 *μ*g/mL	*PE, EtOAc, MeOH*	NM	Cytotoxic activity	NM	[54]

*Coix lacryma-jobi*	Seed, endosperm, and hull	HT-29	0.1–1,000 *μ*g/mL	*Methanolic, hexane*	Phytosterols (campesterol, stigmasterol, and *β*-sitosterol), gamma-linolenic acid (GLA), arachidonic acid (AA), eicosapentaenoicacid (EPA) and docosahexaenoic acid (DHA), linoleic acid	NM	(i) Influence of signal transduction pathways that involve the membrane phospholipids (ii) Enhancement of ROS generation and decrease of cell antioxidant capacity	[166]

*Abutilon indicum*	Leaf	HT-29	210 *μ*g/mL	*Aqueous*	Flavonoids (4H-pyran-4-one, 2,3-dihydro-3,5-dihydroxy-6-methyl, 2-ethoxy-4-vinylphenol, N,N′-dimethylglycine, lup-20(29)-en-3-one, linolenin, 1-mono-, 9-hexadecanoic acid methyl ester, linolenic acid methyl ester), phenolic (amino acids, terpenoids, fatty acids, methyl palmitoleate)	NM	(i) Increase in the levels of reactive oxygen species and simultaneous reduction in cellular antioxidant, mitochondrial membrane loss, DNA damage, and G1/S phase cell cycle arrest	[167]

*Galla rhois*	NM	HCT116, HT-29	12.5, 25, 50, 100, 200 *μ*g/mL	*Aqueous with steaming process*	Gallotannins	Increased contents of gallic acid and ellagic acid	(i) Induced apoptosis through the activation of caspases 3, 8, 9 (ii) Modulated activation of mitogen and protein kinases, p38, and c-Jun NH2-terminal kinase	[168]

*Artemisia annua Linné*	Powder	HCT116	20, 30, 40, 60, 80, 100 *μ*g/mL	*Ethanolic*	Phenolic compounds	Inhibited cell viability and increased LDH release	(i) PTEN/p53/PDK1/Akt signal pathways through PTEN/p53 induce apoptosis (ii) Increased apoptotic bodies, caspase 3 and 7 activation (iii) Regulated cytochrome c translocation to the cytoplasm and Bax translocation to the mitochondrial membrane	[169]

*Nelumbo nucifera* stamen	Powder	HCT116	100, 200, 400 *μ*g/mL	*Ethanolic crude*	NM	NM	(i) Increased the sub-G1 population, mRNA levels of caspases 3 and 8, levels of I*κ*B*α* and caspase 9 (ii) Modulated the Bcl-2 family mRNA expression (iii) Reduced the mRNA levels of NF*κ*B	[170]

Corn silk	NM	LoVo, HCT116	1.25, 2.5, 5, 10, 20 *μ*g/mL	*Aqueous*	Proteins, polysaccharides, flavonoid, vitamins, tannins, alkaloids, mineral salts, steroids	NM	(i) Increase in the Bax, cytochrome c, caspases 3 and 9 levels	[171]

*Lycium barbarum L.*	Powder	HT-29	1, 2, 3, 4, 5 *μ*g/mL	*NM*	Neoxanthin, all-trans-*β*-cryptoxanthin, polysaccharides, carotenoids, flavonoids	NM	(i) Upregulation of p53 and p21 expression (ii) Downregulation of the CDK2, CDK1, cyclin A, and cyclin B expression (iii) Arrest in the G2/M phase of cell cycle	[172]

*Chrysobalanus icaco L.*	Freeze-dried fruit	HT-29	1, 2.5, 5, 10, 20 *μ*g/mL	*Crude ethyl acetate*	Delphinidin, cyanidin, petunidin, and peonidin	NM	(i) Increased intracellular ROS production (ii) Decreased TNF-*α*, IL-1*β*, IL-6, and NF*κ*B1 expressions	[173]

*Zanthoxylum piperitum* De Candolle	Fruit	Caco-2, DLD-1	200 *μ*g/mL	*Aqueous*	NM	NM	(i) Increased the phosphorylation of c-Jun N-terminal kinase (JNK)	[174]

*Celtis aetnensis (Tornab.) Strobl (Ulmaceae)*	Twigs	Caco-2	5, 50, 100, 250, or 500 *μ*g/mL	*Methanolic*	Flavonoid and triterpenic compounds	NM	(i) Increase in the levels of ROS (ii) Decrease in RSH levels and expression of HO-1	[175]

*Rosa canina*	Peel and pulp	Caco-2	62.5, 125, 250, 500 *μ*g/mL	*Total extract (fraction 1), vitamin C (fraction 2), neutral polyphenols (fraction 3), and acidic polyphenols (fraction 4)*	Polyphenols	Decreased production of reactive oxygen species (ROS)	NM	[176]

*Rhazya stricta*	Leaf	HCT116	47, 63, 79, and 95 *μ*g/cm^2^	*Crude alkaloid*	Alkaloids	NM	(i) Downregulated DNA-binding and transcriptional activities of NF*κ*B and AP-1 proteins (ii) Increase in Bax, caspases 3/7 and 9, p53, p21 and Nrf-2 levels (iii) Decrease in expression of ERK MAPK, Bcl-2, cyclin D1, CDK-4, survivin, and VEGF	[177]

*Green coffee*	NM	Caco-2	10-1,000 *μ*g/mL	*NM*	5-Caffeoylquinic acid (5-CQA), 3,5-dicaffeoylquinic acid (3,5-DCQA), ferulic acid (FA), caffeic acid (CA), dihydrocaffeic acid (DHCA), dihydroferulic acid (DHFA)	Reduced viability of cancer cells	NM	[178]

*Flourensia microphylla*	Leaf	HT-29	NM	*Ethanolic and acetone*	Phenolic compounds	NM	(i) Inhibition of IL-8 (ii) Activation of apoptosis by the increment of the Bax/Bcl-2 ratio and expression of TNF family	[179]

^∗^NM: not mentioned.

**Table:** (a) Efficacy of medicinal plants on colon cancer in *in vivo* models

Scientific name	Parts used	Model		Dose	Type of extract	Important compounds	Cellular effect	Mechanisms	References
*Vitis vinifera*	Seed	*In vivo* (murine)	Caco-2	*In vivo*: 400–1,000 mg/kg In vitro: 10–25 *μ*g/mL	*Aqueous*	Procyanidins	(i) Increased crypt depth and growth-inhibitory effects (ii) Inhibited cell viability (iii) Significantly decreased the histological damage score	Reduced MPO (myeloperoxidase) activity	[180]
	Seed	*In vivo*	HT-29, SW480	5 mg/kg	*Aqueous*	NM	NM	Decreased VEGF, TNF, MMP-1, MMP-3, MMP-7, MMP-8, MMP-9, and MMP-13 protein expression	[181]
	Skin	*In vivo*	NM	7.5, 30, 60 *μ*g/mL	*Methanolic*	4′-Geranyloxyferulic acid	NM	NM	[30]
	Seed	*In vivo* (murine)	NM	0.12% *w*/*w*	*NM*	Catechin, epicatechin	NM	(i) Suppressed proliferation, sphere formation, nuclear translocation of *β*-catenin and Wnt/*β*-catenin signaling (ii) Elevated p53, Bax/Bcl-2 ratio, and cleaved PARP and mitochondrial-mediated apoptosis	[31]

*Camellia sinensis*	Leaf	*In vivo* (murine)	HT-29	In vitro: 0, 10, 30, 50 *μ*M In vivo: 1.5 mg per day	*Aqueous*	Catechin, epigallocatechin gallate	1.9-fold increase in tumor endothelial cell apoptosis	Inhibited the ERK-1 and ERK-2 activation, VEGF expression, and VEGF promoter	[182]
		*In vivo* (murine)	HCT116	0.5%	NM	NM	Reduced basement membrane	Inhibition of MMP-9 and VEGF secretion	[183]
		*In vivo* (murine)	Caco-2, HT-29	300 *μ*M	*Aqueous*	Theaflavins (TF-2, TF-3, TF-1)	Induced apoptosis of human colon cancer cells	Inhibition of edema formation correlated to attenuation of COX-2 expression and promoter analysis revealed modulation of NF*κ*B, AP-1, CREB, and/or NF-Il-6 (C/EBP)	[36]
		*In vivo* (murine)	HT115	25 *μ*g/mL	*Hydroethanolic*	Phenolic compounds (p-hydroxyphenyl ethanol, pinoresinol & dihydroxyphenyl ethanol)	NM	Inhibition via reduced expression of a range of *α*5 & *β*1	[184]

*Sasa quelpaertensis*	Leaf	*In vivo*	HT-29, HCT116	0, 100, 200, 300 mg/L	*Ethanolic*	p-Coumaric acid, tricin	Inhibition of colony formation	(i) Nonadherent sphere formation suppressed CD133+ & CD44+ population (ii) Downregulated expression of cancer stem cell markers	[41]

*Anoectochilus*	NM	*In vivo*	CT26	Oral dose of 50 & 10 mg/mouse per day	*Aqueous*	Kinsenoside	Stimulated proliferation of lymphoid tissues	Activation of phagocytosis of peritoneal macrophages	[185]

*Purple-fleshed potatoes*	Fruit	*In vivo*	Colon cancer stem cells	5.0 *μ*g/mL	*Ethanol, methanol, ethyl acetate*	Anthocyanin, *β*-catenin, cytochrome c	Reduction in colon CSCs number and tumor incidence	(i) Increase in cytochrome c levels from p53 status and maybe mitochondria-mediated apoptosis (ii) Suppressed levels of cytoplasmic and nuclear *β*-catenin	[58]

*Phaseolus vulgaris*	Leaf	*In vivo*	HT-29	Nm	*Ethanolic*	Polysaccharides, oligosaccharides	Induction of apoptosis and inhibit proliferation	(i) Inactivation of the retinoblastoma phosphoprotein (ii) Induced G1 arrest (iii) Suppression of NF-jb1 (iv) Increase in EGR1 expression	[59]

*Rosmarinus officinalis* L.	Leaf	*In vivo*	HT-29	SC-RE 30 *μ*g/mL and CA 12.5 *μ*g/mL	*Ethanolic*	Polyphenols (carnosic acid (CA) and carnosol)		(i) Activation of Nrf2 transcription factor (ii) Activated common regulators, such as XBP1 (Xbp1) gene, SREBF1/SREBF2 (Srebp1/2), CEBPA and NR1I2 (Pxr) genes	
	Leaf	*In vivo* (rat)	NM	NM	*Ethanolic*	Rosmanol and its isomers, carnosol, rosmadial, carnosic acid, and 12-methoxycarnosic acid, carnosic acid, carnosol	Interactions with the gut microbiota and by a direct effect on colonocytes with respect to the onset of cancer or its progression	NM	

*Wasabia japonica*	Rhizomes	*In vivo*	COLO 205	5 mg/mL	*Methanolic*	6-(Methylsulfinyl)hexyl isothiocyanate	Anticolon cancer properties through the induction of apoptosis and autophagy	(i) Activation of TNF-*α*, Fas-L, caspases (ii) Truncated Bid and cytochrome c (iii) Decreased phosphorylation of Akt and Mtor (iv) Promoted expression of microtubule-associated protein 1 light chain 3-II and AVO formation	[186]

*Zingiberaceae*	Rhizome	HT-29	HT-29	5 g/kg	*Dichloromethanic*	Turmerone	Suppressed the proliferation of HT-29 colon cancer cells	(i) LDH release (ii) ROS generation (iii) Collapse in mitochondrial membrane potential (iv) Cytochrome c leakage (v) Activation of caspase 9 and caspase 3	[187]

*Panax quinquefolius*	Root	*In vivo* (murine)	NM	30 mg/kg	*Ethanolic*	Ginsenosides (protopanaxadiol or protopanaxatriol)	Attenuated azoxymethane/DSS-induced colon carcinogenesis by reducing the colon tumor number and tumor load	(i) Reduced experimental colitis (ii) Attenuated on AOM/DSS-induced colon carcinogenesis (iii) Proinflammatory cytokines activation (iv) Suppressed DSS (v) Downregulated inflammatory cytokine gene expression	[188]

*Myrtaceae*	Leaf	*In vivo* (murine)	HCT116	100 *μ*g/mL (*in vitro*) 200 and 100 *μ*g/disc (*in vivo*)	*Methanolic*	Phenolics, flavonoids, betulinic acid	Inhibition of tumor angiogenesis	(i) Inhibition of angiogenesis of tube formation on Matrigel matrix and HUVECS migration (*in vitro*) (ii) Decreased nutrient and oxygen supply and consequently tumor growth and tumor size (*in vivo*) (iii) Increased extent of tumor necrosis	[82]

*Spica prunellae*	Leaf	*In vivo*	HT-29	200 mg/mL (*in vitro*), 600 mg/mL (*in vivo*)	*Ethanolic*	Rosmarinic acid	Induction of apoptosis and inhibition of cell proliferation and tumor angiogenesis	(i) Induced apoptosis (ii) Inhibited cancer cell proliferation and angiogenesis STAT3 phosphorylation (iii) Regulated expression of Bcl-2, Bax, cyclin D1, CDK4, VEGF-A, and VEGFR-2 (*in vivo)*	[83]

*Gymnaster koraiensis*	Aerial part	*In vivo* (murine)	NM	500 *μ*mol/kg	*Ethanolic*	Gymnasterkoreaynes B, C, E, 2,9,16-heptadecatrien-4,6-dyne-8-ol	Anti-inflammatory and cancer preventive activities	(i) Significant decrease in expression of COX-2 (ii) Increase in serum IL-6	[189]

*Allium fistulosum*	Edible portions	*In vivo* (murine)	CT26	50 mg/kg b.w.	*Hot water*	p-Coumaric acid, ferulic acid, sinapic acid, quercitrin, isoquercitrin, quercetol, kaempferol	Suppression of tumor growth and enhanced survival rate of test mice	(i) Decreased expression of inflammatory molecular markers (ii) Downregulated expression of MMP-9 and ICAM (iii) Metabolite profiling and candidate active phytochemical components	[190]

*Annona squamosa* Linn	Leaf	*In vivo* (animal)	HCT116	8.98 *μ*g/mL	*Crude ethyl acetate*	Acetogenins (annoreticuin & isoannoreticuin) and alkaloids dopamine, salsolinol, and coclaurine	(i) Inhibited growth and proliferation of tumor cells	Reactive oxygen species (ROS) formation, lactate dehydrogenase (LDH) release, and caspases 3/7, 8, 9 activation	[191]

*Eupatorium cannabinum*	Aerial parts	*In vivo* (murine)	HT-29	25 *μ*g/mL	*Ethanolic*	Pyrrolizidine alkaloids (senecionine, senkirkine, monocrotaline, echimidine)	Cytotoxicity against colon cancer cells	(i) Upregulation of p21 and downregulation of NCL, FOS, and AURKA, indicating reduced proliferation capacity (ii) Mitotic disruption and nonapoptotic cell death via upregulation of Bcl-xL	[96]

*Flacourtia indica*	Aerial parts	*In vivo* (murine)	HCT116	500 *μ*g/mL	*Methanolic*	Phenolic glucoside (flacourticin, 4′-benzoylpoliothrysoside)	Antiproliferative and proapoptotic effects in HCT116 cells	Apoptosis via generation of ROS and activation of caspases (PARP)	[192]

*Sorghum bicolor*	The dermal layer of stalk	*In vivo* (murine)	HCT116 & colon cancer stem cells	>16 and 103 *μ*g/mL	*Phenolic, acetone*	Apigeninidin & luteolinidin	Antiproliferative effect	(i) Target p53-dependent and p53-independent pathways	[97]

*Gleditsia sinensis*	Thorn	*In vivo* (murine)	HCT116	800 *μ*g/mL	*Aqueous*	Flavonoid, lupine acid, ellagic acid glycosides	Inhibited proliferation of colon cancer	(i) Increased p53 levels (ii) Downregulation of the checkpoint proteins, cyclin B1, Cdc2, and Cdc25c	[90]
	Thorn	*In vivo* (murine)	HCT116	600 *μ*g/mL	*Ethanolic*	NM	Inhibitory effect on the proliferation of human colon cancer HCT116 cells	(i) Caused G2/M phase cell cycle arrest	[91]

*Zingiber officinale*	Rhizome	*In vitro/in vivo* (murine)	HCT116	5 *μ*M	*Ethanolic*	6-Paradol, 6- and 10-dehydrogingerdione, 6- and 10-gingerdione, 4-, 6-, 8-, and 10-gingerdiol, 6-methylgingerdiol, zingerone, 6-hydroxyshogaol, 6-, 8-, 10-dehydroshogaol, diarylheptanoids	Inhibitory effects on the proliferation of human colon cancer cells	(i) Arrest of G0/G1 phase (ii) Reduced DNA synthesis (iii) Induced apoptosis	[103]

*Cucumaria frondosa*	The enzymatically hydrolyzed epithelium of the edible	*In vivo* (murine)	HCT116	<150 *μ*g/mL	*Hydroalcoholic*	Monosulphated triterpenoid glycoside frondoside A, the disulphated glycoside frondoside B, the trisulphated glycoside frondoside C	(i) Inhibition at S and G2-M phase with a decrease in Cdc25c (ii) Increase in p21WAF1/CIP	(i) Inhibition the growth of human colon (ii) Apoptosis associated with H2AX phosphorylation and caspase 2	[105]

*Rolandra fruticosa*	Leaf & twigs	*In vivo* (murine)	HT-29	10 and 5 mg/kg/day	*Methanolic*	Sesquiterpene lactone (13-acetoxyrolandrolide)	Antiproliferative effect against human colon cancer cells	(i) Inhibition of the NF*κ*B pathway, subunit p65 (RelA) and upstream mediators IKK*β* and oncogenic K-ras	[106]

*Cydonia oblonga Miller*	Leaf & fruit	*In vivo* (murine)	Caco-2	250–500 *μ*g/mL	*Methanolic*	Phenolic compound (flavonol and flavone heterosides, 5-O-caffeoylquinic acid)	Antiproliferative effect against human kidney and colon cancer cells	(i) Suppression of NF*κ*B activation, activator (AP-1), mitogen-activated protein kinases, namely, PKC, (GFR)-mediated pathways (ii) Cell cycle arrest (iii) Induction of apoptosis, antioxidant, and anti-inflammatory effects	[107]

*Sedum kamtschaticum*	Aerial part	*In vivo* (murine)	HT-29	0–0.5 mg/mL	*Methanolic*	Buddlejasaponin IV	Induced apoptosis in HT-29 human colon cancer cells	(i) Induced apoptosis via mitochondrial-dependent pathway triggered by downregulation of Bcl-2 protein levels, caspase 3 activation, and subsequent PARP cleavage	[109]

*Ganoderma lucidum*	Caps & stalks	*In vivo* (murine)	HT-29	0-0.1 mg/mL	*Triterpene extract (hot water) extract*	Polysaccharides (mainly glucans & glycoproteins), triterpenes (ganoderic acids, ganoderic alcohols, and their derivatives)	Cytokine expression inhibited during early inflammation in colorectal carcinoma	Induced autophagy through inhibition of p38 mitogen-activated kinase and activation of farnesyl protein transferase (FPT)	[193]

*Ginkgo biloba*	Fruit & leaf	*In vivo* (murine)	HT-29	20–320 mg/L	*Aqueous*	Terpene lactones and flavonoid glycosides	Inhibited progression of human colon cancer cells induced HT-29 cell apoptosis	(i) Activation in caspase 3, reduction in Bcl-2 expression, and elevation in p53 expression	[112]

*Rubus occidentalis*	Fruit	*In vivo* (murine)	JB6 Cl 41	25 *μ*g/mL	*Methanolic*	*β*-Carotene, *α*-carotene, ellagic acid, ferulic acid, coumaric acid	Inhibited tumor development	(i) Impaired signal transduction pathways leading to activation of AP-1 and NFB RU-ME fraction	[194]

*Oryza sativa*	Seed	*In vivo* (murine)	HT-29, SW 480, HCEC	100 *μ*g/mL	*Ethyl acetate extract*	Phenolic compound (tricin, ferulic acid, caffeic acid, and methoxycinnamic acid)	Inhibited growth of human colon cancer cells	(i) Induction of apoptosis by enhanced activation of caspases 8 and 3 (ii) Decreased the number of viable SW480 and HCEC cells	[113]

*Cistanche deserticola*	Dried stem	*In vivo* (murine)	SW480	In vivo: 0.4 g/kg/day In vitro: 100 mg/mL	*Aqueous*	Polysaccharides, phenylethanoid glycosides	Decreased mucosal hyperplasia and helicobacter infection	(i) Increased number of splenic macrophages and NK cells (ii) Decreased frequency of hyperplasia and *H. hepaticus* infection of the intestine	[133]

*Rehmannia glutinosa*	NM	*In vivo* (male C57BL6 mice and Sprague-Dawley rats)	CT26	28 mg/kg	*NM*	Catalpol	(i) Inhibited proliferation, growth, and expression of angiogenic markers	(i) VEGF, VEGFR2, HIF-1*α*, bFGF inhibited the expressions of inflammatory factors such as IL-1*β*, IL-6, and IL-8	[143]

*Olea europaea*	Olive mill wastewater	*In vivo* (murine)	NM	NM	*Methanolic*	Hydroxytyrosol	Interferes with tumor cell growth	NM	[195]
	Leaf	*In vivo* (xenograft model) (murine)	HCT116, HCT8	0, 5, 10, 20, 30, 50, and 70 *μ*g/mL	*Phenolic*	Oleuropein and hydroxytyrosol	NM	(i) Activation of caspases 3, 7, and 9 (ii) Decrease of mitochondrial membrane potential and cytochrome c release (iii) Increase in intracellular Ca2+ concentration	[196]

*Ginkgo biloba L.*	Leaf	*In vivo* (rat)	NM	0.675 and 1.35 g/kg	*Methanolic*	Flavonoid glycosides, terpene lactones, and ginkgolic acids	(i) Suppressed tumor cell proliferation, promoted apoptosis, and mitigated inflammation	NM	[197]

*Rhus trilobata Nutt.*	NM	*In vivo* (hamster)	NM	400 mg/kg, 100 mg/kg	*Aqueous*	Tannic acid, gallic acid	Cytotoxic activity	NM	[54]

*Annona diversifolia Saff.*	NM	*In vivo* (mice)	SW-480	1.5, 7.5 mg/kg/day	NM	Laherradurin	Cytotoxic activity	NM	[54]

*A. muricata L.*	NM	*In vivo* (rat)	NM	250/500 mg/kg	*EtOAc*	A, B, and C, and cis- and trans-annomuricin-D-ones	Cytotoxic activity	NM	[54]

*Plumeria acutifolia Poir.*	NM	*In vivo* (hamster)	NM	400 mg/kg/day	*Aqueous*	NM	Cytotoxic activity	NM	[54]

*Lasianthaea podocephala (A. Gray) K. M. Becker*	NM	*In vivo* (hamster)	NM	200 mg/kg/day	*Aqueous*	NM	Cytotoxic activity	NM	[54]

*Flourensia cernua DC.*	NM	*In vivo* (hamster)	NM	350 mg/kg/day	*Aqueous*	Flavonoids, sesquiterpenoids, monoterpenoids, acetylenes, p-acetophenones, benzopyrans, benzofurans	Cytotoxic activity	NM	[54]

*Ambrosia ambrosioides (Cav.) W. W. Payne*	NM	*In vivo* (hamster)	NM	400 mg/kg/day	*Aqueous*	NM	Cytotoxic activity	NM	[54]

*Alnus jorullensis Kunth*	NM	*In vivo* (hamster)	NM	175 mg/kg/day	*Aqueous*	NM	Cytotoxic activity	NM	[54]

*Dimorphocarpa wislizeni (Engelm.) Rollins*	NM	*In vivo* (hamster)	NM	100 mg/kg/day	*Aqueous*	NM	Cytotoxic activity	NM	[54]

*Euphorbia pulcherrima Willd. ex Klotzsch*	NM	*In vivo* (hamster)	NM	200 mg/kg/day	*Aqueous*	NM	Cytotoxic activity	NM	[54]

*Acalypha monostachya Cav.*	NM	*In vivo* (hamster)	NM	400 mg/kg/day	*Aqueous*	NM	Cytotoxic activity	NM	[54]

*Crotalaria longirostrata Hook. & Arn.*	NM	*In vivo* (hamster)	NM	400 mg/kg/day, 350 mg/kg/day	*EtOH-CHCl_3_*	NM	Cytotoxic activity	NM	[54]

*Asterohyptis stellulata (Benth.) Epling*	NM	*In vivo* (hamster)	NM	50 mg/kg/day	*Aqueous*	NM	Cytotoxic activity	NM	[54]

*Acacia constricta A. Gray*	NM	*In vivo* (hamster)	NM	400 mg/kg/day	*Aqueous*	NM	Cytotoxic activity	NM	[54]

*Holodiscus dumosus A. Heller*	NM	*In vivo* (hamster)	NM	350 mg/kg/day	*Aqueous*	NM	Cytotoxic activity	NM	[54]

*Butea monosperma*	Flower	*In vivo* (rat)	HT-29	150 mg/kg	*n-Butanol extract*	Isocoreopsin, butrin, and isobutrin	Free radical scavenging and anticancer activities	NM	[198]

*Taraxacum spp.*	Root	*In vivo* (xenograft murine model)	HT-29, HCT116	40 mg/kg/day	*Aqueous*	*α*-Amyrin, *β*-amyrin, lupeol, and taraxasterol	Induced programmed cell death	NM	[199]

^∗^NM: not mentioned.

**Table:** (b) Other effects of medicinal plants in *in vivo* models

Scientific name	Parts used	Model		Dose	Type of extract	Important compounds	Cellular effect	Mechanisms	References
*Allium sativum*	Root	*In vivo* (murine)	NM	2.4 mL of daily	*Ethanolic*	Allicin, S-allylmercaptocysteine	Significantly suppressed both the size and number of colon adenomas	Enhancement of detoxifying enzymes: SAC and GST activity	[200]

*Olea europaea*	Fruit	*In vivo*	Caco-2	50 *μ*M	*Aqueous*	Phenolic compounds, authentic hydroxyl tyrosol (HT)	(i) Effect of OPE and HT on CB1 associated with reduced proliferation of Caco-2 cells (ii) Increase in CB1 expression in the colon of rats receiving dietary EVOO	Increase in Cnr1 gene expression, CB1 protein levels	[201]
		*In vivo* (murine)	HT115	25 *μ*g/mL	*Hydroethanolic*	Phenolic compounds (p-hydroxyphenyl ethanol, pinoresinol & dihydroxyphenyl ethanol)	NM	Inhibition via reduced expression of a range of *α*5 & *β*1	[184]

*Origanum vulgare* L.	Leaf	*In vivo* (murine)	NM	20, 40, 60 mg·kg^−1^	*Aqueous*	Rosmarinic acid, caffeic acid, flavonoids	Antioxidant status	(i) Increased LPO products and activity of SOD and CAT enzymes and GST and GPx activity (ii) Antioxidant and anticarcinogenic effect	[202]

*Hazelnut*	Skin	*In vivo*	NM	The flow rate 0.21 mL/min and injection volume 9.4 *μ*L	*Aqueous*	Flavan-3-ols, in monomeric and polymeric forms, and phenolic acids	(i) Decreased circulating levels of free fatty acids and triglycerides (ii) Higher excretion of bile acid	Increase of the total antioxidant capacity of plasma	[203]

*Apples and apple juice*	Fruit	*In vivo*	NM	90 mg/L	*Aqueous*	Phenolic acids, flavonoids, tannins, stilbenes, curcuminoids	NM	NM	[204]

*Grifola frondosa*	Fruit	*In vivo* (murine)	HT-29	10 ng/mL	*Aqueous*	Phenolic compounds (pyrogallol, caffeic acid, myricetin, protocatechuic acid, etc.)	Inhibition of TNBS-induced rat colitis	(i) Induced cell cycle progression in G0/G1 phase and apoptotic death	[104]

*Ruta chalepensis*	Leaf	*In vivo* (human)	NM	250 *μ*g/mL	*Ethanolic*	Rutin, gallic acid, catechin hydrate, naringin	Oxidative profile in patients with colon cancer	NM	[205]

*Cannabis sativa*	Dry flower & leaf	*In vivo* (murine)	DLD-1 and HCT116	0.3–5 *μ*M	*Methanolic*	Cannabidiol, phytocannabinoids	NM	(i) Reduced cell proliferation in a CB1-sensitive and AOM-induced preneoplastic lesions and polyps (ii) Inhibition of colorectal cancer cell proliferation via CB1and CB2 receptor activation	[121]

*Melia toosendan*	Fruit	*In vivo* (murine)	SW480, CT26	0, 10, 20, 30, 40, 50 *μ*g/mL	*Ethanolic*	Triterpenoids, flavonoids, polysaccharide, limonoids	NM	(i) Inhibited cell proliferation of SW480 and CT26 by promoting apoptosis as indicated by nuclear chromatin condensation and DNA fragmentation (ii) Induced caspase 9 activity which further activated caspase 3 and poly(ADP-ribose) polymerase cleavage, leading the tumor cells to apoptosis	[123]

*Smallanthus sonchifolius*	Root	*In vivo* (murine)	NM	73.90, 150.74, 147.65, and 123.26 mg/kg	*Aqueous*	Fructans	NM	Reduction incidence of colon tumors expressing altered *β*-catenin	[206]

*Punica granatum*	Peel	*In vivo* (adult male Wistar rats)	NM	4.5 g/kg	*Methanolic*	Gallic acid, protocatechuic acid, cateachin, rutin, ellagic acid, punicalagin	NM	(i) Reduction in TGF-*β*, Bcl-2, EGF, CEA, CCSA-4, MMP-7 and in COX-2, cyclin D1, survivin content (ii) Downregulated expression of *β*-catenin, K-ras, c-Myc genes	[207]

*Linum usitatissimum*	Seed	*In vivo* (male Sprague-Dawley rats)	NM	500 mg/kg	*Alkaline*	Secoisolariciresinol diglucoside, carbohydrates, proteins, and tannins	Reduced the serum fasting glucose levels	Significantly reduced the HbA1c, insulin levels, and proinflammatory cytokines	[208]

*Diospyros kaki*	Fruit	*In vivo* (male CD-1 mice)	NM	15 mg/kg	*Hydroacetone*	Polyphenol	(i) Decreased attenuation of colon length in diarrhea severity (ii) Reduced mortality rate (iii) Reduction of the extent of visible injury (ulcer formation) and of mucosal hemorrhage	Decreased expression of COX-2 and iNOS in the colonic tissue	[147]

*Muntingia calabura*	Leaf	*In vivo* (rat)	NM	50, 250, 500 mg/kg	*Methanolic*	Rutin, gallic acid, ferulic acid, and pinocembrin	Reduction of the colonic oxidative stress, increasing the antioxidants levels possibly via the synergistic action of several flavonoids	NM	[209]

*Portulaca oleracea*	NM	*In vivo* (murine)	HT-29 CSCs	2.25 *μ*g/mL	*Alcoholic*	NM	Regulatory and target genes that mediate the Notch signal transduction pathway	Inhibition of expression of the Notch1 and *β*-catenin genes	[161]

*Aloe vera*	Gel	*In vivo* (murine)	NM	400 mg/kg/day	*Gel*	Polysaccharides	NM	(i) Via inhibition of the cell cycle progression (ii) Induction of cellular factors, such as extracellular signal-regulated kinases 1/2, cyclin-dependent kinase 4, and cyclin D1; on the other hand, PAG increased the expression of caudal-related homeobox transcription factor 2	[210]

*Artemisia annua Linné*	Powder	*In vivo* (xenograft murine model)	HCT116	20, 40 mg/kg/day	*Ethanolic*	Phenolic compounds	NM	(i) Induced apoptosis via PTEN/p53/PDK1/Akt signal pathways through PTEN/p53 (ii) Inhibited cell viability and increased LDH release and apoptotic bodies, caspase 3 and 7 activation, and reduced mitochondria membrane potential (iii) Regulated cytochrome c translocation to the cytoplasm and Bax translocation to the mitochondrial membrane (iv) Regulation of proteins	[169]

*Hordeum vulgare*	Powder	*In vivo* (xenograft murine model)	HT-29	2 g/kg and 1 g/kg	*Aqueous (fermented)*	*β*-Glucan, protein, amino acids, phenolic compounds	NM	(i) Promoted tumor apoptosis by upregulating the mRNA expression of Bax and caspase 3 and downregulating the mRNA expression of Bcl-2 and cyclin D1 (ii) Decreased mRNA expression of Bcl-2 and cyclin D1 (iii) Upregulated expressions levels of Bax and caspase 3	[211]

*Dendrophthoe pentandra*	Leaf	*In vivo* (murine)	NM	125, 250, 500 mg/kg	*Ethanolic*	Quercetin-3-rhamnose	NM	(i) Decreased the levels of IL-22, MPO levels, proliferation of epithelial cells (ii) Inhibited S phase of the cell cycle (iii) Upregulated p53 wild-type gene expression	[212]

*Aquilaria crassna*	Stem, bark	*In vivo* (murine)	HCT116	2,000 mg/kg/day 100, 200 mg/kg	*NM*	Resin and essential oils	NM	NM	[213]

*Berberis integerrima*	NM	*In vivo* (murine)	NM	50 and 100 mg/kg	*Hydroalcoholic*	NM	NM	NM	[214]

*Salix aegyptiaca*	Bark	*In vivo* (murine)	NM	100 and 400 mg/kg	*Ethanolic*	Catechin, catechol, and salicin	NM	Decreased level of EGFR, nuclear *β*-catenin, and COX-2	[215]
